# A Multi-Year Monitoring of Swiss Grain Maize: Which Cropping Factors Influence *Fusarium* Species Incidence and Associated Mycotoxins?

**DOI:** 10.3390/toxins18020065

**Published:** 2026-01-26

**Authors:** Tomke Musa, Karen E. Sullam, Heike Rollwage, Michael Sulyok, Petr Karlovsky, Susanne Vogelgsang

**Affiliations:** 1 Research Group Extension Arable Crops, Agroscope, Reckenholzstrasse 191, 8046 Zürich, Switzerland; susanne.vogelgsang@agroscope.admin.ch; 2 Research Group Molecular Ecology, Agroscope, Reckenholzstrasse 191, 8046 Zürich, Switzerland; karen.sullam@agroscope.admin.ch; 3 Molecular Phytopathology and Mycotoxin Research, Georg-August-University, Grisebachstrasse 6, 37077 Göttingen, Germany; hrollwa@gwdg.de (H.R.); pkarlov@gwdg.de (P.K.); 4 Department of Agricultural Sciences, Institute of Bioanalytics and Agro-Metabolomics, BOKU University, Konrad Lorenz Strasse 20, 3430 Tulln, Austria; michael.sulyok@boku.ac.at

**Keywords:** Gibberella ear rot, Fusarium ear rot, maize, deoxynivalenol, zearalenone, fumonisin, mycotoxin, cropping factor

## Abstract

A complex of *Fusarium* species frequently infects maize, causing root, ear, and stem rot, yield losses, reduced seed quality, and mycotoxin accumulation. To quantify *Fusarium* species composition and mycotoxin contamination, we conducted a first nationwide monitoring in Swiss commercial grain maize over three years (2008–2010), followed by grain maize hybrid experiments across five sites (2011–2013). Samples were analysed for species incidence, fungal DNA, and the mycotoxins deoxynivalenol, zearalenone, and fumonisins. For each field, crop management data were collected. *Fusarium graminearum*, *F. verticillioides*, *F. subglutinans*, and *F. proliferatum* were predominant, and deoxynivalenol was the most frequent toxin, with 55% of the samples exceeding the European pig feed guidance value (0.9 mg kg^−1^). Overall, fumonisin contamination was low: only 11% of samples were above the limit of detection. The year, the length of the growing period, and the timing of the harvest were the principal determinants of *F. graminearum* infection and deoxynivalenol/zearalenone accumulation, whereas other agronomic factors, including crop rotation, soil management, and maturity class, showed only limited or inconsistent effects. Results from this study provide evidence that farmers should avoid long growing periods and late harvests to reduce the risk of high deoxynivalenol/zearalenone content. The maize hybrid experiments confirmed the overriding influence of weather conditions on *Fusarium* species incidence and mycotoxin content, leading to high inter-annual variability. These results highlight the need for standardised, long-term field experiments to disentangle agronomic effects and environmental drivers.

## 1. Introduction

In Europe, maize (*Zea mays* L.) is one of the main arable crops, which is primarily used for animal feed. Maize is frequently infected with a complex of *Fusarium* species [1] causing root, ear, and stem rot. This infection leads to high yield losses and reduced seed quality [2,3,4]. More importantly, these fungi produce mycotoxins, which can cause acute as well as chronic diseases [5]. Severe animal health problems from mycotoxins include a weakened immune system due to deoxynivalenol produced by *F. graminearum* (FG) and *F.culmorum* (FC), reduced fertility due to zearalenone produced by FG, FC, *F. equiseti*, and *F. cerealis* (FCe), or cancer due to fumonisins produced by *F. verticillioides* (FV) and *F. proliferatum* (FPr) [5,6,7]. The death of hundreds of thousands of horses at the end of the 19th and beginning of 20th centuries illustrates the danger of these mycotoxins; they died from so called equine leucoencephalomalacia or ELEM in the United States due to fumonisin contamination of maize. Additionally, pigs and other animals exhibited estrogenic symptoms after consuming maize contaminated with zearalenone from FG [8].

*Fusarium graminearum*, FV, FPr, and *F. subglutinans* (FS) are among the most reported *Fusarium* species isolated from grain maize worldwide [1,3,9,10]. Their incidence not only depends on environmental conditions, such as the prevailing weather during the growing season, but also on agricultural practices. *Fusarium graminearum* produces mainly the mycotoxins deoxynivalenol (DON), 3-acetyl-DON (3ADON), 15-acetyl-DON (15ADON), zearalenone (ZEN), and, depending on the genetic chemotype, also nivalenol [11,12]. In comparison to the cooler and wetter conditions that are more favourable for FG and FS infection, FV and FPr infection and the production of fumonisins (FUM) are more common under hotter and drier conditions. Furthermore, multiple *Fusarium* species may infect grain maize simultaneously and contribute additional mycotoxins, for example, beauvericin, moniliformin, and fusaproliferin, to the overall contamination [13,14].

In 2006, the European Commission established maximum DON, ZEN, and FUM limits for unprocessed cereals for food [15] and guidance values for animal feed [16], which are also applied in Switzerland. The maximum limits for food were updated in 2024 [17], whereas the guidance values for feed remain unchanged. Small-grain cereals and maize have a major economic importance in human and animal nutrition, and the quality as well as the safety requirements are high. As consumers’ and stakeholders’ awareness has increased, so has their demand for grain products free of toxic metabolites. Accordingly, quality standards have increased [18].

In Switzerland, no direct control measures are currently available against maize ear and stem rot, and therefore, only preventive control methods can help reduce the risk of *Fusarium* infection and associated mycotoxin contamination. The development of less susceptible varieties [19,20,21,22] and adaptation of cropping systems is a priority for grain producers. Thus, knowledge about the prevalent *Fusarium* species and mycotoxins is essential to aid in limiting problematic infections. Two Swiss studies [23,24] revealed that up to 16 *Fusarium* species colonised grain maize, and the European guidance values for DON and ZEN were exceeded in up to 68% and 42% of samples, respectively [23]. High FUM concentrations were found in all samples from the southern part of Switzerland (Ticino) [24]. However, these studies were conducted in geographically limited regions and focused primarily on maize grain samples from standardised maize hybrid experiments. In comparison to other European countries, such as Germany [14,25] and Italy [26], no comprehensive study has been conducted in Switzerland on the *Fusarium* species complex in commercial grain maize, which can provide a better understanding of the cropping factors that influence *Fusarium* infections and mycotoxin contamination.

The main objectives of this study were (1) to assemble representative information about the most important *Fusarium* species from commercial grain maize samples in Switzerland during three years of monitoring, (2) to assess the risk of mycotoxin contamination, (3) to identify cropping factors influencing the *Fusarium* infection and mycotoxin contamination, and (4) to develop prevention strategies available for farmers. Furthermore, we aimed to (5) evaluate the potential correlation between *Fusarium* infection or fungal DNA and mycotoxin content. As a complementary and independent component of the study, samples from Agroscope grain maize hybrid experiments conducted between 2011–2013 were analysed separately to specifically assess the effects of maize hybrids and other cropping factors on mycotoxin contamination. These experimental data were not intended for direct comparison with the commercial monitoring dataset but provide data from a more controlled setup.

## 2. Materials and Methods

### 2.1. Monitoring of Commercial Grain Maize (2008–2010)

#### 2.1.1. Field Sample Collections and Processing

**Figure 1:**
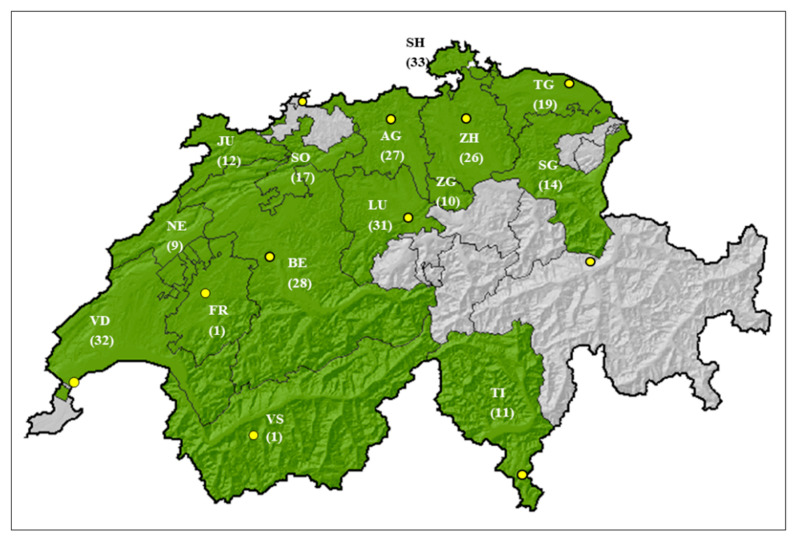
Map of Switzerland (source: Federal Office of Topography, swisstopo). Cantons, from which grain maize samples were received during the sampling period of 2008–2010, are coloured in green. In parentheses: number of received samples per canton. SH = Schaffhausen, TG = Thurgau, JU = Jura, SO = Solothurn, AG = Aargau, ZH = Zurich, SG = St. Gallen, NE = Neuchâtel, BE = Berne, LU = Lucerne, ZG = Zug, VD = Vaud, FR = Fribourg, VS = Valais, TI = Ticino. Yellow circles: location of MeteoSwiss weather stations: Geneva, Sion, Payerne, Berne–Zollikofen, Lucerne, Buchs Aarau, Basel, Zurich Fluntern, Guettingen, Chur, and Lugano.

During the three monitoring years, 271 commercial grain samples (2008: 91; 2009: 97; 2012: 83 samples) were received from Swiss farmers from 15 cantons (Figure 1). Each year, farmers took 1 kg of kernel samples from ten different locations within the field at harvest that were then mixed thoroughly. Subsequently, a subsample of 1 kg from each field was placed in a perforated plastic bag and sent to Agroscope in Zurich. Upon arrival, the samples were immediately dried for three days at 33 °C. For further processing, each grain maize sample was reduced to a working subset using a riffle divider as described in a previous publication [23]. Each working subset consisted of two samples to determine the species incidence (seed health test, SHT), and an additional sample of 250 g was used for mycotoxin and DNA extraction and qPCR analysis. The sample processing, the morphological identification, and ELISA analyses were carried out upon their arrival to the laboratory after each harvest year, while molecular analysis of all samples was performed at the end of the three-year monitoring period. After collection, the samples were stored in −20 °C prior to DNA extraction and qPCR.

For each maize grain sample received, farmers sent a complete questionnaire on various field management properties. It included information on the maize hybrid, previous crop, previous pre-crop, tillage, seedbed preparation, fertilizer regime, planting and harvest time, as well as phytosanitary measures.

#### 2.1.2. *Fusarium* Species Evaluation in Grain Maize Kernels Using a Seed Health Test

To determine the infection rate and incidence of *Fusarium* species, two replicates of each sample were used for a seed health test (each app.: 120–150 kernels) and processed as previously described [23]. In short, kernels were plated on a modified Nash–Snyder medium [27] after surface disinfection in a Chloramin T solution (1%, Riedel-de-Haën, Seelze, Germany). Afterwards, the plates were incubated in the dark at 19 °C for 12 days. Colonies resembling *Fusarium* species were transferred to potato dextrose agar (PDA, Oxoid Ltd., Hampshire, UK) as well as to ‘Synthetic Nutrient Deficient Agar’ (SNA) [28] and incubated (7 days at 19 °C with 12 h dark/12 h near-UV light) before identification based on morphological characteristics [1]. This temperature was chosen to correspond to previously published studies [23,24]. The *Fusarium* incidence was calculated as the average of isolates obtained from 100 maize kernels from two replicates. The relative incidence of *Fusarium* species on maize kernels was calculated as a percentage of the total number of *Fusarium* isolates per year.

#### 2.1.3. Mycotoxin Quantification

These samples came from commercially produced grain maize fields, and ELISA analysis was used for the mycotoxin quantification, because this was the method used at the time at Agroscope. To assess the mycotoxin contamination, the 250 g sample was ground to a fine powder (Retsch Ultra Centrifugal Mill, 1 mm mesh, Verder Catag AG, Haan, Germany). DON, ZEN, and FUM were quantified with commercial ELISA kits (Ridascreen^®^ Fast DON, Ridascreen^®^ Fast Zearalenon and Ridascreen^®^ Fast Fumonisin (sum of B_1_, B_2_, B_3_); R-Biopharm AG, Darmstadt, Germany). For each mycotoxin, 5 g of flour were extracted according to the manufacturer’s instructions. For validation of the ELISA kits, a standardised maize flour (R-Biopharm Rhône Ltd., Glasgow, UK) with known mycotoxin contents was included in each run. The limit of detection was 0.22 mg kg^−1^ for DON, 0.05 mg kg^−1^ for ZEN, and 0.22 mg kg^−1^ for FUM.

#### 2.1.4. *Fusarium* Biomass Determination Using qPCR

To quantify the fungal DNA content, a subsample of the same ground 250 g sample of maize flour used for the mycotoxin analysis was used for the quantitative PCR (qPCR) analysis. A subsample of 0.10 g of flour was filled into 2 mL Eppendorf^®^ safe-lock tubes (Vaudaux-Eppendorf AG, Schönenbuch, Switzerland). DNA from maize flour was extracted using a published CTAB protocol with 2-times downscaled volumes [29]. DNA from pure fungal cultures for standard curves was extracted using a CTAB protocol for fungal mycelia and quantified as described in the same report [29] in which also primers and conditions for the qPCR quantification of FC and FG DNA were described. The primers and conditions for qPCR quantification of FPr and FV DNA were described in Nutz et al. [30]; for FS, in Becker et al. [31]; and for *F. poae* (FP), in Beule et al. [32].

#### 2.1.5. Weather Data (Commercial Grain Maize Monitoring)

During each monitoring year, weather data (average temperature (°C) and sum of precipitation (mm)) from 11 MeteoSwiss weather stations (Geneva, Sion, Payerne, Berne–Zollikofen, Basel, Buchs Aarau, Lucerne, Zurich/Fluntern, Chur, Güttingen, and Lugano) were obtained. The weather stations were located in regions where most of the samples were collected. They covered different regions of Switzerland. To compare the monthly variation of the measured parameters at the different weather stations during each monitoring year, long-term averages from MeteoSwiss (“climate normals” between 1981 and 2010) of precipitation and temperature were used. Climate normals are mean parameter values collected over 30 years to describe the typical local climate of each month at a given site. In the monitoring period of 2008–2010, a month was considered warmer or cooler to the climate normals if the temperature deviated by more than ±0.5 °C and was wetter/drier if the precipitation deviated by more than ±10 mm relative to the climate normals. 

The sum of precipitation for these 11 weather stations was calculated for each single month (June, July, August, September, and October) and for the whole period (June–October) in each monitoring year.

### 2.2. Grain Maize Hybrid Experiments (2011–2013)

#### 2.2.1. Field Sample Collection and Processing

Grain maize hybrid experiments of Agroscope were conducted during three years at five different locations in Switzerland: Zurich-Reckenholz (canton Zurich), Ellighausen (Thurgau), Goumoëns (Vaud), Delley (Fribourg), and Cadenazzo (Ticino). The experimental design, including the cob sampling procedure, was followed as previously described [24]. All fields were ploughed before maize hybrids were sown. Two maize hybrids out of each maturity class (early, mid-early, and mid-late) were considered. At the experimental sites Zurich-Reckenholz, Ellighausen, Gouemoëns, and Delley, samples from the following maize hybrids and maturity classes were investigated: Birko and Stuard (2011/2012), respectively, Laurinio (2013), classified as early maturity; NK Top (2011/2013), respectively, NK Cooler (2012) and Ricardinio, classified as mid-early maturity; as well as Cassilas and DK3240, classified as a mid-late maturity class. At Cadenazzo, the selection of grain maize hybrids and maturity classes was different from the other locations, and samples from four maize hybrids were examined: PR38A24 and DKC5267 (early maturity) as well as PR34B39 and Maxxis (2011/2012), respectively, Kassandras (2013) (mid-late maturity). Sample sizes from these experiments were the same as those used in the commercial grain maize monitoring. Delley was the only location with smaller sampling amounts (parts of the sample were required for feed-specific analysis), and working subsamples consisted of two samples with 100 kernels for species incidence and 50 g for mycotoxin analysis. Fertilisation as well as pest and weed control were carried out according to agricultural practice for integrated production in Switzerland. In all hybrid experiments, maize hybrid plants were only exposed to natural *Fusarium* infections. The processing, morphological analysis, and mycotoxin analyses of the samples were carried out upon their periodic arrival to the laboratory each year.

#### 2.2.2. Incidence of *Fusarium* Species in Maize Kernels Using a Seed Health Test

To determine the infection rate of *Fusarium* species, the same method was used as described above (Section 2.1.2).

#### 2.2.3. LC-MS/MS-Based Quantification of Mycotoxins

For the quantification of mycotoxins, maize flour samples from the maize hybrid experiments were sent to the Institute of Bioanalytics and Agro-Metabolomics, Department of Agrobiotechnology (IFA-Tulln), in Austria. The samples were analysed with an LC-MS/MS-based multi-method covering more than 300 analytes at the time of analysis [33]. Five-g aliquots of the samples were extracted for 90 min with 20 mL of acetonitrile/water/acetic acid (79:20:1, *v*/*v*/*v*) on a GFL 3017 rotary shaker (GFL, Burgwedel, Germany). The extracts were diluted 1 + 1 with acetonitrile/water/acetic acid (20:79:1, *v*/*v*/*v*), and 5 μL of the diluted extracts were injected without further pre-treatment.

Analysis was performed on a QTrap 5500 MS/MS system (AB Sciex, Framingham, MA, USA) and a 1290 series UHPLC system (Agilent Technologies, Waldbronn, Germany). Chromatographic separation was obtained at 25 °C on a Gemini^®^ C_18_ column (150 × 4.6 mm, i.d.; 5 μm, particle size) equipped with a C_18_ security guard cartridge (4 × 3 mm, i.d.) (all from Phenomenex, Torrance, CA, USA). Elution was carried out in binary gradient mode using an acidified acetonitrile/water gradient. ESI-MS/MS was performed in the scheduled selected reaction monitoring (sSRM) mode both in positive and negative polarities in two separate chromatographic runs. Further instrumental details are listed in Malachova et al. [33].

Two sMRMs per analyte were obtained to confirm each positive analyte’s identification. In addition, the retention time and the intensity ratio had to agree within 0.03 min and 30% rel., respectively, with an authentic standard. Mycotoxin quantification was based on serial dilution of a multianalyte stock solution. Results were corrected for apparent recoveries obtained during method validation for maize. Method performance parameters are listed in Malachova et al. [33]. The accuracy of the method is verified on a routine basis by participation in an interlaboratory testing scheme organised by BIPEA (Gennevilliers, France). Satisfactory z-scores between −2 and 2 have been obtained for >96% of >2300 of the results submitted so far.

#### 2.2.4. Weather Data for Grain Maize Hybrid Experiment

Data of the parameter average temperature (°C) and sum of precipitation (mm) were obtained from four MeteoSwiss weather stations (Payerne, Güttingen, Zurich-Affoltern, and Magadino) representative of the experimental sites Delley, Ellighausen, Goumoëns, Zurich-Reckenholz, and Cadenazzo. Monthly precipitation sums and temperature means were calculated for each experimental year (2011, 2012, and 2013). As above, the weather data were compared with climate normals from MeteoSwiss, and a month was considered warmer or cooler to the climate normals if the temperature deviated by more than ±0.5 °C and wetter/drier if the precipitation deviated by more than ±10 mm relative to the climate normals.

### 2.3. Statistical Analysis for Commercial Maize Monitoring and Grain Maize Hybrid Experiments

All statistical analyses were performed in R Studio (R version 4.4.3) [34]. For each dataset (monitoring (2008–2010) and hybrid experiments (2011–2013)), a factor analysis of mixed data (FAMD) was executed separately, and the packages “FactoMineR” and “factoextra” were used for this analysis. The FAMD uses both quantitative and categorical variables to analyse a dataset through a principle component method [35]. The similarities between samples are examined considering mixed types of variables, and the relationship between all variables, including quantitative and categorical variables, can be analysed.

To characterise mycotoxin contamination and examine whether particular agronomic factors were associated with different mycotoxin levels, we then divided DON, ZEN, and FUM data into categorical levels based on each measured mycotoxin content. We used the detection limits of the ELISA^®^ RIDASCREEN assays, together with EU guidance values for mycotoxin contamination in animal feed [16], to define the classification thresholds for DON, ZEN, and FUM. Although ELISA assays do not provide the same sensitivity as LC-MS/MS, they enable us to divide all commercial and grain maize hybrid mycotoxin datapoints into “no”, “low”, “medium”, “high”, and “very high” categories (Table 1) according to the classification criteria for each mycotoxin. The number of samples in each category as well as their proportion (%) is shown in the Supplementary Materials, Table S1.

To estimate if and to which extent factors are responsible for driving samples into particular mycotoxin categories, a v-test was conducted, using the “catdes” function of the “FactoMineR” package (R version 4.4.3). For categorical variables, a chi-square test and a one-way analysis of variance (ANOVA) on quantitative variables was performed. To reduce the possibility of Type I errors due to multiple comparisons, a more stringent alpha value (α = 0.001) was used. Results of the v-test are presented as heat maps.

To analyse the correlations between incidence and biomass of *Fusarium* species (seed health test and qPCR data, respectively) and the associated mycotoxins, Spearman’s correlation coefficient was calculated using the “corr.test” function from the “psych” R package (R version 4.4.3). Prior to the analysis, seed health data were arscin (square root)-transformed, while qPCR and mycotoxin data were log-transformed to meet the assumption of the normal distribution of the residuals. Correlations were deemed significant at alpha ≤ 0.05 after Holm adjustment of *p*-values to account for multiple comparisons. In the graphs, only correlation coefficients of significant relationships are shown. The size and colour of the circle represent the strength of the relationship. The axes are arranged based on hierarchical clustering (“hclust” function in R from the “stats” package, R version 4.4.3) using the default complete linkage method. For the dataset of maize hybrid experiments, only incidence of *Fusarium* species based on seed health tests were available.

To analyse overall yearly differences in *Fusarium* species incidence, *Fusarium* species biomass (DNA), and mycotoxin contamination, a one-way ANOVA with transformed data was used for both datasets (commercial grain maize and grain maize hybrid experiments). If the assumption of normal distribution of residuals after the transformation was not met, the non-parametric Kruskal–Wallis test was used.

#### 2.3.1. Analysis of *Commercial Grain Maize Monitoring* (2008–2010)

The categorical variables included in the FAMD were previous crop, previous pre-crop, maturity class, type of soil management, sowing time, harvest time, presence or absence of a cover crop, use or no use of a seed dressing, and the year of harvest (Table 2). As too little information was received on the occurrence of hail and European corn borer (*Ostrinia nubilalis*) attacks, these factors were excluded from statistical analysis. The type of production system was not included as a factor, because 98% of the fields were managed according to PEP (proof of ecological performance). No fungicides are registered against maize ear and stem rot in Switzerland, and no insecticides in the field are applied. The quantitative factors included growing period, mycotoxin concentrations, species quantification based on qPCR, and species incidence based on a seed health test for the following species: FG, FV, FPr, FS, FP, and FC (Table 2).

#### 2.3.2. Analysis of the *Grain Maize Hybrid Experiments* (2011–2013)

The dataset of the *grain maize hybrid experiments* was analysed by FAMD as described above, and the quantitative and categorical variables used are compiled in Table 3. No fungicides or insecticides were applied. The same categorisation for the different mycotoxin levels was used as described in the previous section (Section 2.3, Table 1), and a v-test was conducted to estimate if and to which extent variables are responsible for a mycotoxin category using the “catdes” function in the “FactoMineR” package (R version 4.4.3). Results are also presented in heat maps, showing the association between the variables and the mycotoxin categories. To identify maturity class effects on DON, ZEN, and FUM concentrations, fold changes relative to early maturity classes were calculated using log_2_ transformation. FUM was log_10_-transformed to meet the assumptions of normality. Differences among maturity classes were assessed using a one-way ANOVA followed by a Tukey HSD post hoc test in R (packages: dplyr, tidyr, ggplot2, purrr, broom, and readxl, R version 4.4.3).

As no overlapping grain maize hybrids were used at the Cadenazzo site compared with the four sites North of the Alps, this experimental site was excluded from the analysis.

## 3. Results

### 3.1. Commercial Grain Maize Monitoring (2008–2010)

#### 3.1.1. *Fusarium* Species Spectrum and Fungal Incidence

**Figure 2:**
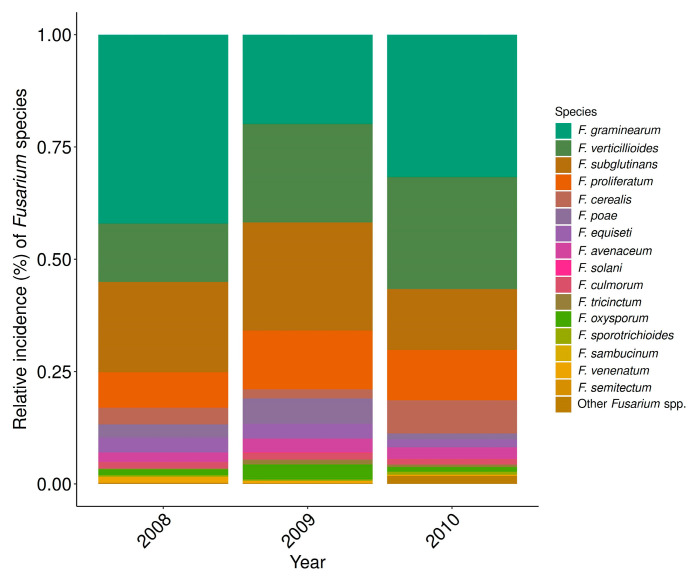
Variation in the frequency pattern of identified *Fusarium* species in infected grain maize kernels examined in Switzerland over the three monitoring years: 2008 (*n* = 91), 2009 (*n* = 97), and 2010 (*n* = 83). Data from seed health tests.

From the 271 maize kernel samples, 6017 *Fusarium* isolates were morphologically identified. Overall, the incidence of *Fusarium* species on kernels in 2008 (14%) was significantly lower (*p* < 0.001) than in 2009 (22%) and in 2010 (31%). Sixteen *Fusarium* species were identified, and the most frequent species throughout this monitoring were FG, FV, FS, and FPr. Other less prevalent *Fusarium* species were, in descending order, FCe, FP, *F. equiseti*, *F. avenaceum*, *F. solani*, FC, *F. oxysporum*, *F. tricinctum*, *F. sporotrichioides*, *F. sambucinum F. semitectum*, and *F. venenatum* (Figure 2; Supplementary Materials, Table S2). Although misidentification among closely related *Fusarium* species is possible, we are confident in our species assignment, as this study focuses on the most dominant and important species.

The frequency pattern of the dominant *Fusarium* species composition varied considerably throughout the years. In 2008, FG was clearly predominant with a relative incidence of 41% in the infected maize kernels, whereas in 2009, FV, FS, and FG occurred in almost equal parts (21, 22, and 22%, respectively). In 2010, FG and FV occurred more frequently than FS and FPr (Figure 2; Supplementary Materials, Table S2).

Consistent with the total *Fusarium* incidence, qPCR also showed the lowest total *Fusarium* DNA content in 2008 (2.1 µg g^−1^ maize grain flour) and a significantly higher content in 2009 (8.0 µg g^−1^) and 2010 (39 µg g^−1^) (*p* < 0.001) (Supplementary Materials, Table S2). Maximum measured fungal biomass was observed in samples from 2010 with 1.5 µg g^−1^ for FG, 8.8 µg g^−1^ for FV, 0.54 µg g^−1^ for FPr, 0.73 µg g^−1^ for FS, and 1.4 µg g^−1^ for FC.

**Figure 3:**
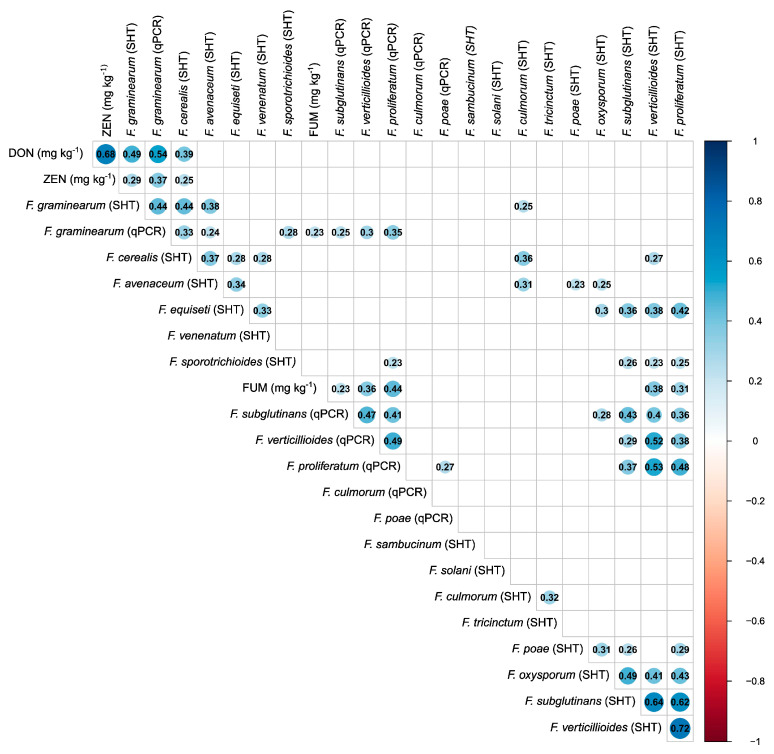
Significant Spearman rank correlation coefficients between the incidences of fungal species (%) based on seed health tests (SHTs), mycotoxin concentration, and *Fusarium* DNA content from *commercial grain maize samples* obtained between 2008 and 2010 (*n* = 271). The size and colour of the circle represent the strength of the relationship. The axes are arranged based on hierarchical clustering (hclust function in R) using the default complete linkage method. *Fusarium* DNA (qPCR) was measured from six *Fusarium* species: *F. graminearum*, *F. verticillioides*, *F. proliferatum*, *F. subglutinans*, *F. culmorum*, and *F. poae*. DON = deoxynivalenol, ZEN = zearalenone, FUM = fumonisins.

The correlation between the qPCR data and the seed health test (SHT) data were positive and of similar magnitude for FV (ρ = 0.52, *p* < 0.001), FPr (ρ = 0.48, *p* < 0.001), FG (ρ = 0.44, *p* < 0.001), and FS (ρ = 0.43, *p* < 0.001). No significant correlation between qPCR data and SHTs was found for the two species FP and FC. *Fusarium verticillioides* and FPr incidence showed the strongest positive correlation (FV-FPr ρ = 0.72, *p* < 0.001), while for FG and FCe, a moderate positive correlation (ρ = 0.44, *p* < 0.001) was observed (Figure 3).

#### 3.1.2. Occurrence of Mycotoxins

In this monitoring study, a substantial yearly variability was also observed for DON contamination. In 2008 and 2010, 64% and 71% of the samples, respectively, exceeded the European guidance value for complementary and complete feeding stuff for pigs (0.9 mg kg^−1^), whereas in 2009, the proportion was markedly lower at 30%. For ZEN, the number of samples that exceeded the European guidance value for complementary and complete feeding stuff for sows and fattening pigs was generally lower, although the same yearly variability seen for DON was noticed (Table 4). The analysis across all samples over the three years showed a significantly lower mean DON and ZEN content in 2009 than in the two other years (*p* < 0.001). A strong positive correlation was found between DON and ZEN contamination (ρ = 0.68, *p* < 0.001).

A moderate positive correlation was observed between DON contamination and FG biomass (determined as DNA concentration) and FG incidence (seed health test), with comparable strengths for FG biomass (ρ = 0.54, *p* < 0.001) and for FG incidence (ρ = 0.49, *p* < 0.001). Only weak but significant correlations were found between ZEN contamination and FG incidence (ρ = 0.29, *p* < 0.001), FG biomass (ρ = 0.37, *p* < 0.001), and FCe incidence (ρ = 0.25, *p* < 0.001).

Throughout the entire three-year monitoring period, the number of samples contaminated with FUM was very low. Only 11% of all samples showed quantifiable FUM levels, and only six samples (2%) exceeded the FUM guidance level for complementary and complete feeding stuff for pigs, horses, rabbits, and pet animals (5 mg kg^−1^) [16]. Four out of these six samples originated from the southern canton, Ticino.

Despite the low numbers of samples, FUM contamination correlated stronger with FPr biomass, determined as concentration of DNA, than with FV biomass (ρ = 0.44, ρ = 0.36, respectively, *p* < 0.001).

#### 3.1.3. Effects of Cropping Factors on Mycotoxin Contamination

**Figure 4:**
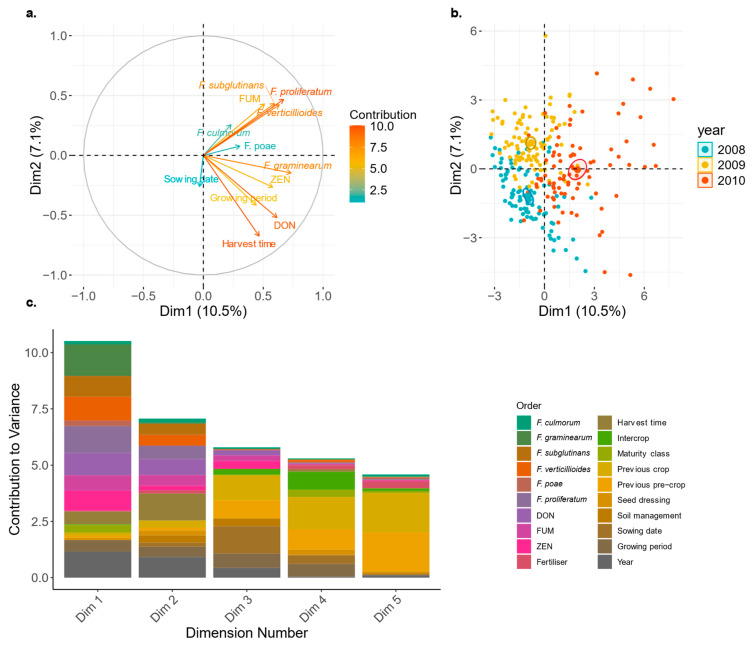
(**a**–**c**). Analysis of the *Swiss commercial maize monitoring (2008–2010)* data (**a**). Correlation circle of quantitative variables from factor analysis of mixed data (FAMD) with the 1st and 2nd dimensions of the principal components shown. Arrows show variable direction and strength, with length and colour indicating their contribution magnitude to the two dimensions. (**b**). Individual datapoints from the analysis are shown on the first two dimensions and are coloured according to the monitoring year (ellipses correspond to 95% confidence intervals around the centroid of each year). (**c**). Variance decomposition showing contributions of the variables to the first five dimensions of the FAMD analysis. DON = deoxynivalenol, ZEN = zearalenone, FUM = fumonisins.

Based on the factor analysis of mixed data (FAMD), 18% of the total variance could be explained by its first two dimensions and 33% by its first five dimensions, underlining the complexity of the dataset. The correlation circle of quantitative variables showed a strong association of DON content, harvest time, and growing period and to a smaller extent, to FG DNA and ZEN content (Figure 4a). When all cropping factors are analysed together with the mycotoxin data using the FAMD, FUM content also showed a strong association with the FUM-producing *Fusarium* species FV and FPr and the non-FUM-producing FS and a negative correlation with DON and ZEN content (Figure 4a).

We found a distinct clustering of the individual datapoints according to the different monitoring years (Figure 4b). Variance decomposition emphasised the strong impact of the factor year, harvest time, and growing period on the first two dimensions. Conversely, the cropping factors soil management, previous crop, previous pre-crop, cover crop, maturity class, sowing date, fertiliser, and seed dressing contributed just to the third to fifth dimension, revealing therefore a smaller impact on *Fusarium* DNA contents analysed by qPCR and mycotoxin contamination (Figure 4c).

**Figure 5:**
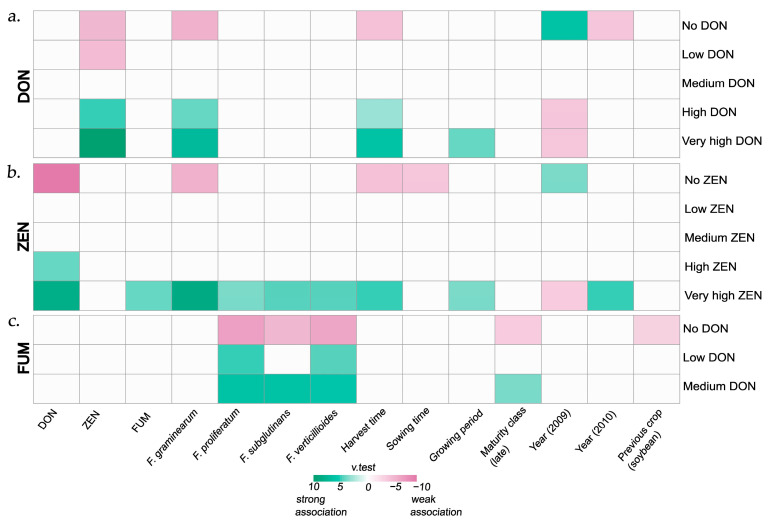
(**a**–**c**). Associations based on v-test results between factors in *the commercial grain maize monitoring* dataset and the mycotoxin level categories of (**a**). deoxynivalenol (DON), (**b**). zearalenone (ZEN), and (**c**). fumonisins (FUM). The intensity of the colour represents the strength of the association calculated from the v-test score. Green colour indicates a positive association, pink colour, a negative association. For information on the categorisations “no”, “low”, “medium”, “high”, and “very high”, see Table 1 (Section 2.3).

The three heat maps (Figure 5a–c) based on v-test results (Supplementary Materials, Table S5) show the associations between each explanatory variable and the defined categories, including “no”, “low”, “medium”, “high”, and “very high” mycotoxin levels (see Section 2.3, Table 1, for information on mycotoxin level categorisation).

The heat map for DON (Figure 5a) indicates that samples from 2009 were positively associated (coloured in green) with the category “no DON”. Fields with samples in the “no DON” category were harvested, on average, 7.5-days earlier than the overall harvest date. In contrast, samples in the “very high DON” category showed an association with a later harvest date (+12 days above the overall mean) and a longer growing period (+9 days above the overall mean) (Supplementary Materials, Table S5). *Fusarium graminearum* biomass and ZEN contamination correlated positively with the “high” and “very high DON” categories. Only samples with low ZEN contamination were associated with the “low DON” category, and no variables were found to be significantly related to “medium DON” (Figure 5a).

For ZEN, primarily samples from the “no” and “very high” ZEN categories were significantly associated with certain variables (Figure 5b); samples from 2009, those with a slightly earlier sowing date (−3 days compared to the overall average sowing date), and those with an average earlier harvest date (−3 days) were more likely to belong to the category “no ZEN”. Samples from the year 2010 and those with a delayed harvest (on average, + 11 days compared to the overall mean) and a longer growing period (on average, + 8 days compared to the overall mean) were more likely to fall in the “very high ZEN” category (Supplementary Materials, Table S5). In addition, samples in the “very high ZEN” category were also associated with a higher FG, FV, FPr, and FS amount as well as higher DON and FUM content than the samples’ overall means of these values. Samples in the “no ZEN” category, on the other hand, had a significantly lower FG biomass and DON content than the overall mean (Figure 5b). Other cropping factors, including soil management, previous crop, previous pre-crop, seed dressing, cover crop, and fertiliser, were not associated with any of the DON and ZEN categories.

Unlike the DON and ZEN categories, the FUM categories were not associated with year, harvest date, sowing date, or growing period (Figure 5c). However, late maturing hybrids were overrepresented in the category “medium FUM” and underrepresented in the category “no FUM”. Samples with previous crop “soybean” were negatively associated (coloured in pink) with “no FUM”, indicating a potential increased risk of FUM contamination with soybean as previous crop (Supplementary Materials, Table S5). Samples with higher FV, FPr, and FS biomass were assigned to the “medium FUM” category or the “low FUM” category for high FV and FPr biomass, while samples with lower biomass of these species were associated with the “no FUM” category (Figure 5c).

#### 3.1.4. Weather Conditions (2008–2010)

Weather conditions differed substantially during the three years of monitoring. In 2008, June was warmer and drier than the long-term climate normals at the eleven selected weather stations, but in the following months, July until October, the sum of precipitation was in general higher than climate normals. In contrast, in the 2009 season, higher sums of precipitation were measured only in July, but August, September, and October showed unusually dry conditions with up to less than 35% precipitation than the long-term average and up to 2 °C warmer temperatures than the climate normals (Supplementary Materials, Figure S1). The 2010 season started with a warm and dry June and July, but in August, weather conditions changed to cooler temperatures and more humid conditions. Although temperatures stayed mainly cooler during September and October, less precipitation was observed than for long-term norm values.

### 3.2. Grain Maize Hybrid Experiments (2011–2013)

#### 3.2.1. *Fusarium* Species Spectrum and Fungal Incidence

All fields of the *grain maize hybrid experiments* were ploughed, and two hybrids from each different maturity class, early, mid-early and mid-late, were selected (Section 2.3, Table 3). Analysis of these experiments showed similar *Fusarium* species diversity as in the Swiss monitoring of commercial grain maize. Up to 15 different *Fusarium* species were morphologically identified, and the predominant species were FG, FV, FPr, and FS again. *Fusarium sambucinum* was not observed in this dataset.

**Figure 6:**
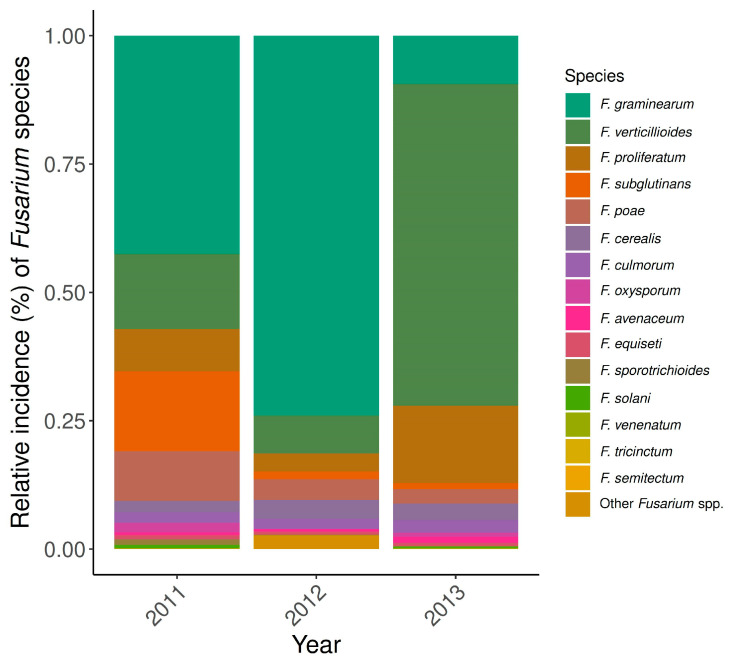
*Grain maize hybrid experiments* conducted in 2011, 2012, and 2013 (*n* = 216) show yearly variation in the frequency pattern of identified *Fusarium* species in infected kernels. Data are shown for the experimental sites Zurich-Reckenholz (ZH), Ellighausen (TG), Goumoëns (VD), and Delley (FR). The experimental site Cadenazzo (TI) was excluded as maize hybrids differed from those sown at the other sites.

The frequency of *Fusarium* species was equally characterised by a strong annual variability. In 2011 and 2012, FG was clearly the most frequent *Fusarium* species, and it composed of 42 and 73% of the isolates, respectively, whereas in 2013, FV was the dominant species, accounting for 63% of the isolates (Figure 6; Supplementary Materials, Table S3).

The total *Fusarium* species incidence based on the percentage of kernels infected over all sites was only 9% in 2011, which was significantly lower than the infection rates of 18% and 28% found in 2012 and 2013, respectively (*p* < 0.001).

**Figure 7:**
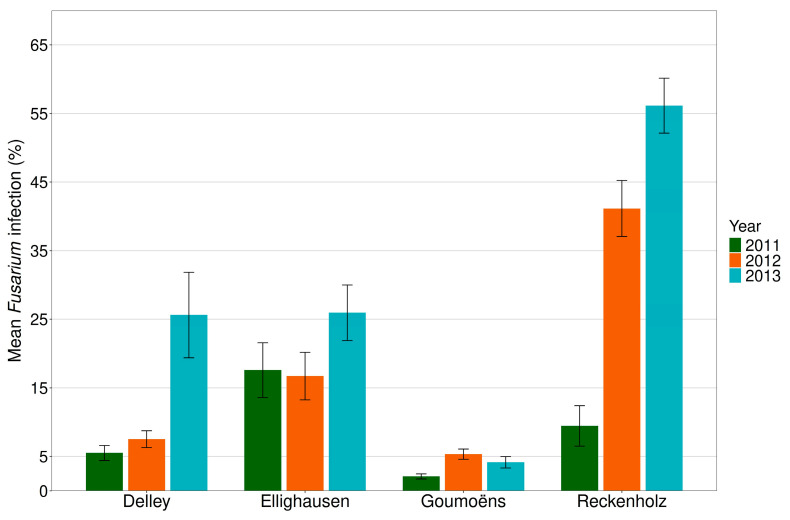
Mean *Fusarium* incidence in analysed kernels of the *grain maize hybrid experiments* throughout three different years (2011, 2012, and 2013) at the four experimental sites Delley (FR), Ellighausen (TG), Goumoëns (VD), and Zurich-Reckenholz (ZH) (*n* = 216). The site Cadenazzo (TI) was excluded as maize hybrids differed from those sown in the other sites.

The mean *Fusarium* incidence differed between years and sites (Figure 7). The lowest disease pressure was observed at the experimental site Goumoëns, showing mean *Fusarium* incidences of 2% (2011), 5% (2012), and 4% (2013). In contrast, the highest disease pressure was found in 2013 at the experimental site Reckenholz (56%) followed by Ellighausen (26%) and Delley (26%). In 2012, higher mean *Fusarium* incidences were found at the two more eastern-located experimental sites Reckenholz (41%) and Ellighausen (17%), compared to the two more western-located sites Delley (8%) and Goumoëns (5%). *Fusarium* composition varied between the four different experimental sites. In 2011 and 2012, FG was the most prevalent species across all experimental sites, while FV dominated in 2013, except for Goumoëns, where no kernels with FV infection were detected (Supplementary Materials, Figure S4).

#### 3.2.2. Occurrence of Mycotoxins

Mycotoxin contamination also showed yearly variability (Table 5). In accordance with the *Fusarium* incidence, DON contamination was significantly higher in 2012 than in 2013 and 2011 (*p* < 0.001). The percentage of samples exceeding the European guidance value for DON was 51% and 30% in the years 2012 and 2013, respectively. In 2011, this proportion was only 14%. The overall contamination with ZEN and FUM was lower than DON at the four experimental sites, but significant differences were again observed between years (Table 5). Only one single sample exceeded the European FUM guidance level of complementary and complete feeding stuff for pigs, horses, rabbits, and pet animals (5 mg kg^−1^). The number of samples which exceeded the limit of detection for FUM was highest in 2013 with 51% of the samples followed by 17% in 2012 and 11% in 2011.

At the southern experimental site Cadenazzo, which was excluded from the overall analysis due to non-congruent maize hybrids, FUM contamination was detected above the limit of detection in all samples across all three years. In addition, 27% of the samples exceeded the EU guidance value for FUM. We also observed that in 2011 and 2012, the relative incidence of FPr was higher than FV (Supplementary Materials, Tables S9–S11 and Figure S6).

**Figure 8:**
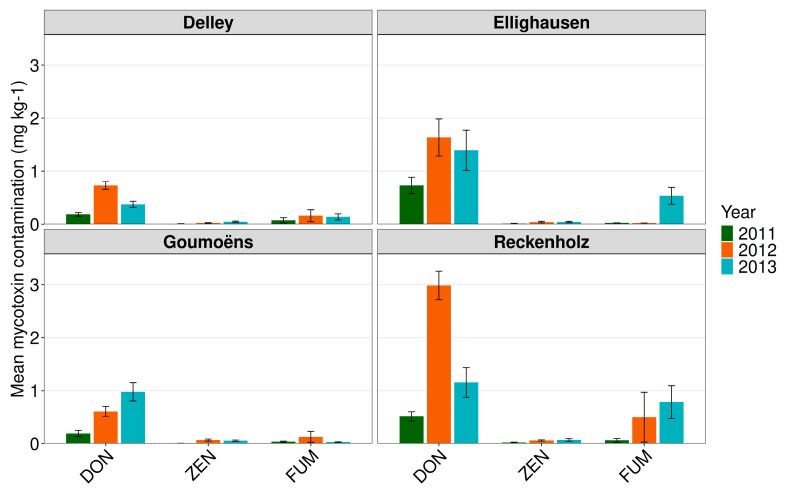
Mean mycotoxin content (mg kg^−1^) in analysed kernels of the *grain maize hybrid experiments* in 2011, 2012, and 2013 at the four experimental sites Delley (FR), Ellighausen (TG), Goumoëns (VD), and Zurich-Reckenholz (ZH). DON = deoxynivalenol, ZEN = zearalenone, FUM = fumonisins. The site Cadenazzo (TI) was excluded as maize hybrids differed from those sown at the other sites.

Deoxynivalenol was the most predominant mycotoxin in all three years and across all four experimental sites, but the contamination level differed between the years and sites. The experimental site Reckenholz had higher DON contamination than Goumoëns and Delley but was not significantly different from Ellighausen. Ellighausen showed higher contamination than Delley, yet did not differ significantly from Goumoëns, particularly in 2012 (Figure 8) (*p* < 0.001). As for the *Fusarium* incidence, overall mycotoxin contamination was lower at the experimental sites Goumoëns and Delley in comparison with Ellighausen and Reckenholz. Fumonisin contamination was mainly observed in 2013 at the two eastern-located sites Ellighausen and Reckenholz and in 2012 at Reckenholz (Figure 8). There was no significant difference between any of the maturity classes, independent of the year or the experimental site (Supplementary Materials, Figure S5).

#### 3.2.3. Weather Conditions (2011–2013)

Total precipitation from June to October was higher in 2012 than in the other two experimental years at all four sites (Supplementary Materials, Figure S2). This difference was particularly striking at the Reckenholz site, where approximately 40% less rain was measured during the same period in 2011 and 2013. In 2012, the mean monthly temperatures were higher in June and August across all sites, whereas July 2011 was cooler and wetter compared with the long-term climate normals (MeteoSwiss) but followed by a dry period until October. In 2013, July and October experienced higher-than-average temperatures and exceptionally high rainfall in October. Inter-annual weather variation, particularly regarding precipitation, was most pronounced at the Reckenholz site (Supplementary Materials, Figure S2).

#### 3.2.4. Effects of Cropping Factors on Mycotoxin Contamination in the *Grain Maize Hybrid Experiments*

To examine the impact of cropping factors on the dominant *Fusarium* species and mycotoxin contamination, data were also subjected to FAMD. The analysis revealed that 27% of the total variance could be explained by its first two dimensions and 56% by its first five dimensions, underlying again the complexity of the dataset. Here, the experimental setup, however, was more standardised than the monitoring dataset because all fields were ploughed and maize hybrids with similar maturity classes and harvest dates were selected.

**Figure 9:**
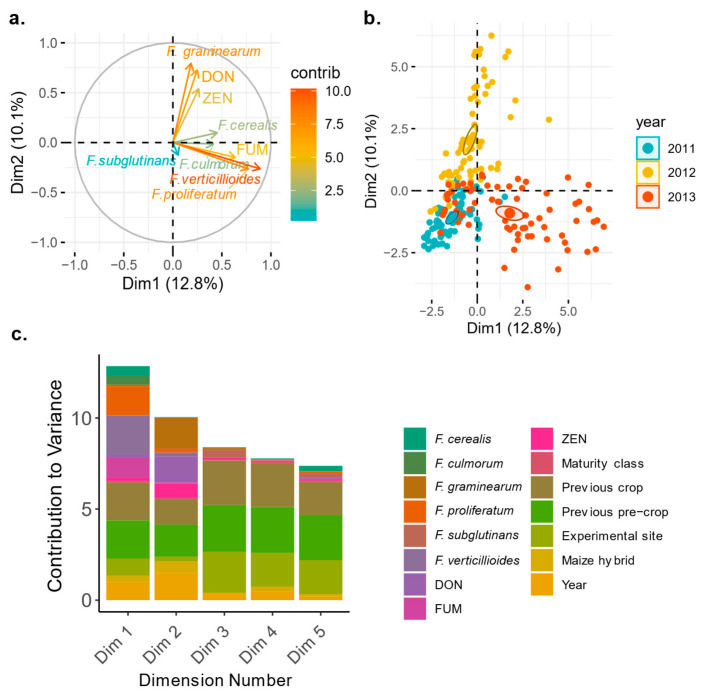
(**a**–**c**). Analysis of the grain maize hybrid experiment data. (**a**). Correlation circle of quantitative variables from factor analysis of mixed data (FAMD) with the 1st and 2nd dimensions of the principal components shown. Arrows show variable direction and strength, with length and colour indicating their contribution magnitude to the two dimensions. (**b**). Individual datapoints from analyses are shown on the first two dimensions and coloured according to the monitoring year (ellipses correspond to 95% confidence intervals around the centroid of each year) to the qualitative variable of year. (**c**). Variance decomposition showing contributions of the variables to the first five dimensions of the FAMD analysis. DON = deoxynivalenol, ZEN = zearalenone, FUM = fumonisins.

The correlation circle of quantitative variables indicates an even more pronounced association of DON content with FG incidence and ZEN content (Figure 9a) when compared to the commercial grain maize monitoring data (Figure 4a). Again, there was a strong correlation between FUM content and the FUM-producing species FV and FPr, along with a negative correlation between FUM and DON/ZEN content (Figure 9a). However, unlike the monitoring data, the variance decomposition from the grain maize hybrid experiment highlighted not only the significant influence of the year (Figure 9b) but also the effects of previous pre-crop, previous crop, and experimental site on the first two dimensions (Figure 9c).

**Figure 10:**
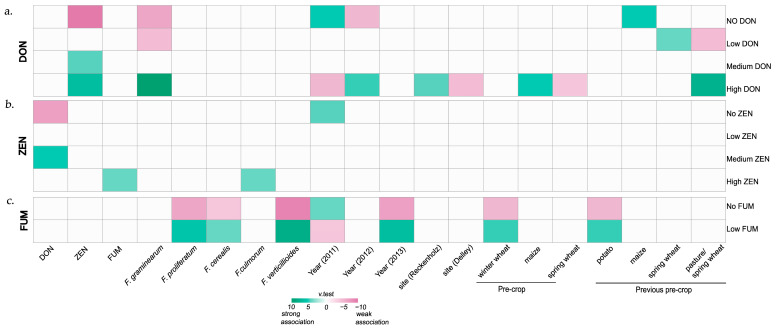
(**a**–**c**). Associations based on v-test results between factors in the *grain maize experiments* data and the mycotoxin level categories of (**a**). deoxynivalenol (DON), (**b**). zearalenone (ZEN), and (**c**). fumonisins (FUM). The intensity of the colour represents the strength of the association calculated from the v-test score. Green colour indicates a positive association, pink colour, a negative association. For information on the categorisations “no”, “low”, “medium”, “high”, and “very high”, see Table 1 (Section 2.3).

To identify variables significantly associated with the defined mycotoxin categories (Section 2.3, Table 1), heat maps were generated based on v-test results (Supplementary Materials, Table S6). Unlike the maize monitoring dataset, none of the samples from the hybrid experiment had mycotoxin values that classified them in the “very high” mycotoxin category for DON, ZEN, or FUM; therefore, this category is not displayed in the heat maps (Figure 10a–c).

For DON, the heat map indicates that samples categorised as “no”, “low”, and “high DON” were primarily associated with specific variables (Figure 10a): samples from 2011 and those with a previous pre-crop maize were positively (green) associated with the “no DON” category. Samples with a previous pre-crop spring wheat were more likely to be assigned to the category “low DON”, while those with a previous pre-crop of spring wheat/pasture were negatively (pink) associated with this category. In contrast, samples harvested in 2012, those from the experimental site Reckenholz as well as samples with a previous crop maize or previous pre-crop spring wheat/pasture, were strongly associated with the category “high DON”. Conversely, at the experimental site Delley in the year 2011, samples with a previous crop spring wheat were negatively associated with the “high DON” category. Furthermore, samples classified as “high DON” had higher FG incidence and ZEN contents relative to the overall mean, whereas those in the “no” and “low DON” categories had significantly lower FG and ZEN levels (Supplementary Materials, Table S6).

In contrast to DON, cropping factors did not have a significant effect on ZEN categories (Figure 10b). Only samples from 2011 were more frequently associated with the “no ZEN” category. In fact, the association of samples with ZEN categories was rather driven by co-occurring mycotoxins: samples in the “no ZEN” category had generally low DON contents, while those in the “medium ZEN” category were linked to higher DON contents. Samples in the “high ZEN” category showed increased FUM contents and FC incidence compared with the overall mean.

Based on the defined mycotoxin categories, all samples’ values fell into the “no” or “low FUM” category (Figure 10c). As FUM contamination was higher in 2013 than in the other two years, samples from 2013 were positively associated with the “low FUM” category. In addition, samples with a previous crop winter wheat as well as samples with previous pre-crop potatoes were positively associated with the “low FUM” category.

In summary, the *grain maize hybrid experiments* showed a strong influence of the year and experimental site on mycotoxin contamination. They also highlighted the importance of cropping history, particularly previous crop and previous pre-crop, with the strongest effects observed for DON (Figure 10a–c). In contrast, based on the FAMD analysis, no significant associations were found between contamination levels and maturity class (Supplementary Materials, Table S6).

## 4. Discussion

Our study presents results of a very first nationwide, multi-year monitoring of commercial grain maize samples (2008–2010) and experiments with grain maize hybrids (2011–2013) in Switzerland. Consistent with previous research [23,36,37,38], we confirmed a high diversity of *Fusarium* species in infected grain maize kernels. In the following sections, we compare our results with previous reports and discuss management practices that may help mitigate mycotoxin contamination in Swiss grain maize.

### 4.1. Species Composition and Yearly Variability

Out of the detected *Fusarium* species, FG, FV, FPr, and FS were the most dominant species across all years and sites. In Europe, FG and FC are known to be the main species causing Gibberella ear rot and FV, FPr, and FS causing Fusarium ear rot in maize [39]. The observed variability in the *Fusarium* species composition also aligns with findings from maize monitoring studies conducted by Görtz et al., Scauflaire et al., and Pfordt et al. [25,36,37]. Furthermore, other studies on maize showed that during seasons with high precipitation and moderate temperatures, FG was the prevailing *Fusarium* species leading to high DON and ZEN contamination [40], whereas drought stress followed by rainfall increased the risk of FUM contamination [41]. Weather conditions during the six years of the reported grain maize investigations varied substantially. The two dry and hot seasons in 2009 and 2013 led to a shift in *Fusarium* species composition from FG dominance towards the more heat-tolerant species FV and FPr, a result which has also been noted by Cao et al. [42].

### 4.2. Mycotoxin Dynamics Depending on Weather Conditions and Species Co-Occurrence

We observed a shift in the proportion of grain maize samples exceeding the European DON guidance value for pigs (0.9 mg kg^−1^): In 2009 and 2013, 30% of the samples exceeded this guidance value compared with 64%, 71%, and 51% in 2008, 2010, and 2012, respectively. In 2011, despite the 42% relative FG incidence in the analysed kernels, mean DON contamination across the four experimental sites was unexpectedly low, and only 14% of the samples exceeded the DON guidance value. These results can be explained by the weather conditions that were favourable for FG infection in July 2011 but not in the following months until harvest, which were dry and less suitable for DON synthesis. Dalla Lana et al. found that temperature (15–30 °C) and high relative humidity (>80% or >90%) during the period after silking (1–21 days) increased the risk of DON contamination [43]. Other studies on wheat and Fusarium head blight (FHB) caused by FG documented an increase in DON contamination due to late-season rainfall or high humidity without increasing FHB infection [44,45].

Similarly, substantial exceedances of the European guidance value for ZEN (0.25 mg kg^−1^ for sows and fattening pigs) occurred in 2008 (54%) and 2010 (32%). Especially in 2008, the sum of precipitation during September and October was higher than long-term normals (MeteoSchweiz, 1991–2010), which favours ZEN synthesis [46]. These results demonstrate that the contamination of Swiss grain maize kernels with DON and ZEN poses a considerable risk to feed safety, since the majority of Swiss maize production is designated for animal feed, while only a minor fraction is utilised for human consumption [47]. The potential hazard to livestock is further exacerbated when grain maize is used directly on farms without prior analysis for mycotoxin contamination [48,49].

In 2009, August and September temperatures were up to 2 °C higher than climate normals, with reduced rainfall from August to October. In 2013, a warm, dry period lasted from July to September, followed by an exceptional rainy October. Both years showed a clear shift in *Fusarium* incidence towards FV and FPr, but 2009, unlike 2013, showed no increase in FUM contamination. The humid conditions before harvest in 2013 could explain the higher proportion of samples with FUM contents above the limit of detection [50,51].

With the exception of the southern experimental site in Cadenazzo, the overall level of FUM contamination across the four other experimental sites was low despite high FV incidence, which was consistent with the two former Swiss studies [23,24]. This could be due to the co-occurrence of different *Fusarium* species. Picot et al. reported that prior infection with FG increased FV DNA in maize kernels but without elevating FUM levels. In maize kernels co-inoculated with FG-FV, FV DNA was consistently higher than in single FV inoculation. As FV can grow saprophytically and fumonisins are not considered virulence factors in the process of infection, the authors concluded that FG colonisation may facilitate FV infection [52]. Similarly, Sherif et al. observed a stimulation of FV growth when co-infected with or following FG infection, hypothesising that through FG infection, nutrients are released, which stimulates the growth of FV [53]. They also reported that fumonisins inhibit FG growth in vitro [53] and suggested that FUM accumulated in grains shed on the ground protect the grains from competitors of FUM producers. Based on our datasets with solely natural infection and sample assessments after harvest, it was not possible to determine which *Fusarium* species colonised maize plants first and if one species was inhibited or rather stimulated by another. Nevertheless, mycotoxins clearly co-occur [48,49,54]. Another reason for the low FUM contamination could be the same as the findings of Stępień et al., who stated that 20% of the investigated strains did not produce FUM, possibly reflecting deletions or other defects within the FUM gene cluster [55]. Moreover, the FUM concentration in our study may have been underestimated due to the presence of masked forms. In fact, in recent years, analytical methods have been further developed to routinely analyse masked forms of different mycotoxins [56,57]. Future studies should employ advanced detection techniques, while isolated FV strains could be tested for their ability to produce FUM.

### 4.3. Impact of Agronomic Factors on Mycotoxin Accumulation in Grain Maize

In terms of agronomic factors, our FAMD analysis revealed that harvest date and the length of the growing period were the most critical determinants of DON and ZEN contamination in the *commercial maize monitoring study* from 2008–2010. Extended field exposure to fungal infection due to delayed harvest was associated with high DON and ZEN levels. These findings support previous studies linking late-season exposure to increased fungal growth and mycotoxin production [58,59,60].

Other agronomic factors such as tillage, crop rotation, seed treatment, cover crops, fertiliser, and hybrid maturity class did not show a significant association with our DON and ZEN categories in the *commercial maize monitoring* dataset. For FUM, however, previous crop soybean and late maturity class showed higher risk for FUM contamination. These findings must, however, be interpreted with caution due to the small number of FUM-contaminated samples in this dataset. The lack of consistent association between the other agronomic factors and DON/ZEN contamination may reflect the dominant-year effect driven by overall weather conditions and high variability in environmental and management conditions in our nationwide dataset.

For our *grain maize hybrid experiments*, all fields were ploughed, yet 60% of the samples exceeded the EU guidance value for DON for pigs, indicating no reliable reduction in DON contamination through tillage. While reduced DON levels following ploughing have been reported in small grain cereals [12,61,62,63], evidence in maize remains inconsistent. Some studies observed a reduction in FG infection and DON [25,64], whereas others, including our results, found no significant effect [65,66,67]. For example, Mansfield et al. reported lower DON levels under moldboard tillage compared with samples under direct sowing [68]. In contrast, Borràs-Vallverdú et al. [69] found higher DON concentrations in intensively tilled maize compared with no-till plots. They attributed this to soil crusting, which promotes water pooling and higher air humidity conducive for fungal growth, as well as a reduced population of earthworms that indirectly help minimise fungal infection and mycotoxin production in maize by accelerating the degradation of *Fusarium* biomass in plant residues serving as an inoculum source [69].

In both datasets, samples with high FUM contamination were consistently found in ploughed fields, suggesting that tillage may not reduce contamination as reported in previous studies [70,71,72]. Although the number of highly contaminated samples in our nationwide monitoring was small, all originated from ploughed fields, as did all samples from the maize hybrid experiments, including those from the southern site of Cadenazzo, which showed the highest contamination levels. This suggests that ploughing has no reducing effect against FUM contamination. The reason for this finding could be explained by the long-term persistence of certain *Fusarium* species. Vegetative compatibility tests have shown that *FV* and *FPr* can survive for at least 630 days in both surface and buried maize residues [71], and also, FG was found to persist in buried host tissue, given that the tissue integrity is maintained [72]. Therefore, ploughing may have brought infected crop residues back to the surface.

The more standardised *maize hybrid experiments*, in which all fields were sown with selected grain maize hybrids and managed with uniform ploughing and similar harvest dates, were conducted to identify differences between the effect of the selected maturity classes and the previous crops. FAMD analysis did not reveal any significant influence of the maturity class on DON content, but a notable association in crop rotation was found besides a strong year and site impact. Samples from fields with previous crop maize were more likely to show elevated DON content, especially in combination with previous pre-crop pasture/spring wheat. On the contrary, samples with previous or previous pre-crop spring wheat were clearly associated with low DON content. No association between other previous crops, such as potatoes, sugar beets, winter wheat, and canola, was found with any of the defined DON categories. Although these findings suggest a significant association between crop rotation and DON content, this effect may be confounded by an unbalanced distribution of previous pre-crop and previous crop across the experimental sites.

In the literature, the impact of crop rotation on mycotoxin contamination in maize is controversial. Pfordt et al. and Mansfield et al. reported no effect of crop rotation on DON content [25,68], whereas Hooker et al. [65] found no significant impact of tillage systems. In their study, the presence of maize in the crop rotation within the preceding two years or with winter wheat as previous crop appeared to exert a greater influence on DON and fumonisin B_1_ content in maize than the elevated surface residue levels due to reduced tillage [65].

Several studies emphasised the importance of choosing appropriate previous crops, especially soybeans, to reduce the risk of FUM contamination. Abbas et al. found less FUM contamination in maize with a soybean–maize rotation [73]. Although soybean is generally associated with little or no mycotoxin contamination, occasional presence of *Fusarium* species cannot be excluded and may contribute to high DON and FUM contamination, as reported by Rodrigues et al. [74]. In our nationwide monitoring, fields with soybean as previous crop were less likely to fall into the “no FUM” category. It is possible that *Fusarium* species colonising soybean residues may have served as a source of inoculum for the subsequent maize crop.

Hybrid maturity class had no influence on mycotoxin contamination in our *grain maize hybrid experiments* according to the DON, ZEN, and FUM categories. In the nationwide monitoring dataset, planting late maturing maize hybrids resulted overall in higher FUM contents. This is in line with the study of Battilani et al. [75]; however, they also stated that the duration of the drying process is even more important than the maturity class. The number of FUM-contaminated samples in Switzerland was too small for a conclusive interpretation of the influence of agronomic factors on FUM contamination.

Given the complexity of *Fusarium* infection and mycotoxin contamination in grain maize, standardised field experiments are essential for developing targeted strategies to mitigate *Fusarium*-related risks and associated mycotoxins. Balanced designs across maize hybrids, maturity classes, crop rotations, and tillage systems are required to untangle factor effects from the strong year and site influence, allowing for a clearer evaluation of individual factors by minimising variability based on uncontrolled influences. Complementary to controlled experiments, large-scale and long-term monitoring is crucial for detecting changes in *Fusarium* occurrence and population composition, particularly in response to climate change. As temperature and humidity patterns shift, *Fusarium* prevalence and mycotoxin production profiles are expected to change, potentially increasing the risk of maize contamination [76].

Since we found only a few agronomic factors consistently influencing mycotoxin production, additional measures, such as the use of resistant hybrids, are needed to reduce mycotoxin contamination. Apart from resistance genes [21], features of the cob structure, such as the number and the coverage of husk leaves, also serve as a barrier to the infection [77], explaining why insect-resistant maize expressing Cry protein is less infected with FV [78] and contains substantially less FUM than non-transgenic hybrids [79]. Hence, breeding programmes aiming at reducing FUM contamination should also consider useful cob characteristics and insect resistance.

## 5. Conclusions

Our multi-year study, including a 3-year *commercial grain maize survey* and 3-year *grain maize hybrid experiments*, confirms a high diversity of *Fusarium* species in Swiss grain maize, of which FG, FV, FPr, and FS predominate. Mycotoxin contamination, especially DON and ZEN, showed strong year-to-year variability, primarily driven by weather conditions. Hot and dry seasons favoured FV and FPr, while wet, moderate conditions increased DON and ZEN risks caused by FG. Despite high FV presence, FUM levels remained low, possibly due to non-producing strains or co-infection dynamics. Agronomic factors such as harvest date and growing period length influenced DON and ZEN levels, while soil tillage showed no consistent impact. Overall, DON, ZEN, and FUM pose a considerable feed safety risk in Swiss grain maize, underlining the importance of regular mycotoxin monitoring.

To address the growing challenge of *Fusarium*-related risks under climate change, future research should focus on standardised field experiments, molecular diagnostics, and long-term surveillance. An earlier harvest, selection of resistant hybrids, and diligent crop rotation remain practical measures for mitigation, but more precise and adaptive strategies will be essential to safeguard maize quality in the coming decades.

## Figures and Tables

**Table 1:** Mycotoxin categories used for the statistical analysis of both datasets (2008–2010, commercial grain maize dataset (*n* = 271); 2011–2013, grain maize hybrid experiments) based on mycotoxin levels and EU guidance values (=GVA) for mycotoxin contamination in animal feed.

	Defined Range of Categories (GVA = Guidance Value, LOD = Limit of Detection)
DON	
no DON	0 to <0.22 mg kg^−1^
low DON	≥0.22 (LOD ELISA) to <0.9 mg kg^−1^
medium DON	≥0.9 (GVA for pigs) to <2 mg kg^−1^
high DON	≥2 (GVA for calves < 4 months, lambs, and kids) to <5 mg kg^−1^
very high DON	≥5 mg kg^−1^ (complementary and complete feeding stuff with above-mentioned exceptions)
ZEN	
no ZEN	0 to <0.05 mg kg^−1^
low ZEN	≥0.05 (LOD ELISA) to <0.1 mg kg^−1^
medium ZEN	≥0.1 (GVA for piglets/gilts) to <0.25 mg kg^−1^
high ZEN	≥0.25 (GVA sows and fattening pigs) to <0.5 mg kg^−1^
very high ZEN	≥0.5 mg kg^−1^ (GVA for calves, dairy cattle, sheep, and goats)
FUM	
no FUM	0 to <0.22 mg kg^−1^
low FUM	≥0.22 (LOD ELISA) to <5.0 mg kg^−1^
medium FUM	≥5 (pigs, horses, rabbits, and pets) to <20 mg kg^−1^
high FUM	≥20 mg kg^−1^ (poultry, calves < 4 months, lambs, and kids) to <50 mg kg^−1^
very high FUM	≥50 mg kg^−1^ (adult ruminants > 4 months and mink)

**Table 2:** Categorical and quantitative variables of the *commercial grain maize monitoring* dataset (*n* = 271) included in the factor analysis of mixed data (FAMD). The number of samples included in the analysis are shown in parentheses.

**Categorical Variables**	
Previous crop ^1^	wheat (98), barley (43), maize (39), pasture (32), sugar beet (28), canola (15), potato (6), soybean (5), sunflower (5)
Previous pre-crop ^2^	wheat (83), maize (45), canola (41), pasture (37), barley (22), sugar beet (20), potato (14), peas (4), sunflower (5)
Maturing class	mid-early (123), early (78), late (66), na ^3^ (4)
Soil management	plough (191), reduced tillage ^d^ (80)
Sowing time	Julian day
Harvest time	Julian day
Mineral fertiliser	with (198), without (73)
Seed dressing	with (188), without (45), na ^3^ (38)
Cover crops	without (202), with (69)
Harvest year	2008 (91), 2009 (97), 2010 (83)
**Quantitative variables**	
Growing period	number of days between sowing and harvest
Mycotoxin concentrations	DON mg kg^−1^, ZEN mg kg^−1^, FUM mg kg^−1^
Species abundance (qPCR)	*F. graminearum*, *F. verticillioides*, *F. proliferatum*, *F. subglutinans*, *F. poae*, *F. culmorum*

^1/2^ If a previous crop or previous pre-crop species was below 3 samples, these samples were excluded from FAMD analysis. Previous crops: cabbage (1), carrot (2), chicory (1), onion (1), pea (1), rice (1), and strawberries (1). Previous pre-crops: beetroot (1), carrot (2), onion (1), rice (1), oat (2), strawberries (1), and no information provided (2). ^3^ na = no information provided; ^d^ includes no-till samples (10).

**Table 3:** Categorical and quantitative variables of the *grain maize hybrid experiment dataset* included in the factor analysis of mixed data (FAMD). The number of samples is in parentheses.

**Categorical Variables**	
Previous crop	spring wheat (66), sugar beet (24), canola (36), maize (30), potato (24), winter wheat (18), pasture/potato (18)
Previous pre-crop	soybean (54), winter/spring wheat (36), spring wheat (30), maize (18), pasture (18), winter wheat (18), potato (18), pasture/spring wheat (18), sugar beet (6)
Maturing class	early (24), mid-early (24), mid-late (24)
Grain maize hybrid	Birko, Stuard (2011/2012)/Laurinio (2013); NK Top (2011/2013)/NK Cooler (2013); Ricardinio, DKC3420, Cassillas
Harvest time	Julian day
Sites	Zurich-Reckenholz (ZH), Ellighausen (TG), Delley (FR), Goumoëns (VD)
Harvest year	2011 (72), 2012 (72), 2013 (72)
**Quantitative variables**	
Mycotoxin concentration	DON mg kg^−1^, ZEN mg kg^−1^, FUM mg kg^−1^
Species incidence (SHT) ^1^	*F. graminearum*, *F. verticillioides*, *F. proliferatum*, *F. subglutinans*

^1^ SHT = seed health test.

**Table 4:** Mycotoxin data from the *Swiss commercial grain maize monitoring for 2008–2010*, *n* = 271. Mean deoxynivalenol and zearalenone contents (mg kg^−1^) as well as the percentages of grain maize samples exceeding their corresponding European guidance values. Means of the years with the same letters are not significantly different according to a one-way ANOVA (*p* < 0.001).

Year	Deoxynivalenol			Zearalenone		
	Mean Content (mg kg^−1^) ± 95% Confidence Interval		% Samples Above Guidance Value ^1^	Mean Content (mg kg^−1^) ± 95% Confidence Interval		% Samples Above Guidance Value ^2^
2008	2.93 ± 0.51	a	64	0.20 ± 0.04	a	54
2009	0.84 ± 0.14	b	30	0.14 ± 0.06	b	5
2010	4.13 ± 0.80	a	71	0.54 ± 0.12	a	32

^1^ European guidance value of 0.9 mg kg^−1^ for DON for complementary and complete feeding stuff for pigs [16]. ^2^ European guidance value of 0.25 mg kg^−1^ for ZEN for complementary and complete feeding stuff for sows and fattening pigs [16].

**Table 5:** Mycotoxin data from the *grain maize hybrid experiments* (2011–2013), *n* = 216 (site Cadenazzo was excluded). Mean deoxynivalenol, zearalenone, and fumonisin contents (mg kg^−1^) as well as the percentages of grain maize samples exceeding the corresponding European guidance values. Values from years sharing the same letter are not significantly different from each other (α = 0.05).

Year	Deoxynivalenol			Zearalenone			Fumonisins		
	Mean Content (mg kg^−1^) ± 95% Confidence Interval		% Samples Above Guidance Value ^1^	Mean Content (mg kg^−1^) ± 95% Confidence Interval		% Samples Above Guidance Value ^2^	Mean Content (mg kg^−1^) ± 95% Confidence Interval		% Samples Above Guidance Value ^3^
2011	0.41 ± 0.05	c	14	0.01 ± 0.002	b	0	0.05 ± 0.02	b	0
2012	1.5 ± 0.16	a	51	0.05 ± 0.01	a	4	0.2 ± 0.12	b	1
2013	0.98 ± 0.13	b	30	0.05 ± 0.01	a	4	0.37 ± 0.09	a	0

^1^ European guidance value of 0.9 mg kg^−1^ for DON for complementary and complete feeding stuff for pigs [16]. ^2^ European guidance value of 0.25 mg kg^−1^ for ZEN for complementary and complete feeding stuff for sows and fattening pigs [16]. ^3^ European guidance value of 5 mg kg^−1^ for FUM for complementary and complete feeding stuff for pigs, horses rabbits, and pet animals [16].

## Data Availability

The original contributions presented in this study are included in the article/Supplementary Materials. Further inquiries can be directed to the corresponding author.

## Supplementary Materials

The following supporting information can be downloaded at: https://www.mdpi.com/article/10.3390/toxins18020065/s1, Figure S1: Monthly temperature (C°) and monthly sum of precipitation (mm) during the *commercial grain maize monitoring* years in Switzerland (2008–2010) compared to the MeteoSwiss monthly “climate normals” (1981–2010) of eleven MeteoSwiss weather stations; Figure S2: Monthly average temperature (°C) and monthly sum of precipitation (mm) during the *grain maize hybrid experiments* (2011–2013) at the four experimental sites Delley (FR), Ellighausen (TG), Goumoëns (VD), and Zurich-Reckenholz (ZH) compared to the MeteoSwiss monthly “climate normals” (1981–2010) from representative weather stations. Payerne represents experimental sites Delley and Goumoëns, Güttingen represents experimental site Ellighausen and Zurich-Reckenholz represents experimental site Zurich-Reckenholz; Figure S3: Significant Spearman rank correlation coefficients between the incidences of fungal species based on seed health test (SHT) and mycotoxins concentration from *grain maize hybrid experiments* at the four experimental sites Delley, Ellighausen, Goumoëns and Zurich-Reckenholz (2011–2013). The experimental site Cadenazzo was excluded due to non-congruent maize hybrids; Figure S4: Mean *Fusarium* incidence and composition pattern in analysed kernels of the *grain maize hybrid experiments* from early, mid-early, and mid-late maturing maize hybrids throughout three different years (2011, 2012, 2013) at the four experimental sites Delley (FR), Ellighausen (TG), Goumoëns (VD), and Zurich-Reckenholz (ZH), (*n* = 216). The site Cadenazzo (TI) was excluded as maize hybrids differed from those sown in the other sites; Figure S5: Log_2_ fold changes in mycotoxin content (DON, FUM, and ZEN) across maize maturity classes mid-early and mid-late relative to the maturity class early (reference, log_2_ fold change = 0) at the four experimental sites Delley, Ellighausen, Goumoëns and Reckenholz during 2011–2013. Bars represent mean maturity-driven effects after removal of site and year effects. The absence of significance symbols indicates that no statistically significant differences among maturity classes were detected (Tukey HSD, *p* < 0.05); Figure S6: Monthly mean temperature (°C) and monthly sum of precipitation (mm) at the MeteoSwiss weather station Magadino during 2011–2013 compared with MeteoSwiss “climate normals” ((1981–2010); red: temperature (°C), blue: precipitation (mm)). The weather station Magadino is representative for the grain maize hybrid experimental site Cadenazzo (Ticino); Table S1: Mycotoxin categories used for the statistical analysis of both data sets (2008–2010 commercial grain maize data set (*n* = 271); 2011–2013 grain maize hybrid experiments) based on mycotoxin levels and EU guidance values (=GVA) for mycotoxin contamination in animal feed and the number of samples in a category as well as their proportion (%); Table S2: Morphologically identified *Fusarium* species throughout the *commercial maize grain monitoring* in Switzerland 2008–2010, listed according to their relative frequency (%) in the infected grain maize kernels. Mean incidence (%) based on a seed health test and mean DNA (ng g^−1^) shown for the four most frequent *Fusarium* species (*n* = 271); Table S3: Morphologically identified *Fusarium* species throughout the *grain maize hybrid experiments* in Switzerland 2011–2013, listed according to their relative frequency (%) in the infected grain maize kernels. In addition, mean incidence (%) based on a seed health test of the four most prevailing *Fusarium* species in the single years is also indicated (*n* = 216). As no overlapping grain maize hybrids were sown at the site Cadenazzo compared with the four sites North of the Alps, this experimental site was excluded from the analysis; Table S4: Difference in the sum of precipitation (mm) in the years 2011 and 2013 compared with 2012 at the *grain maize hybrid experimental sites* Delley, Ellighausen, Goumoëns and Zurich-Reckenholz. Listed MeteoSwiss weather stations are representative for the experimental sites; Table S5: Detailed results of FAMD- analysis of *commercial grain maize monitoring* data set, 2008–2010, (*n* = 271). Association of the different factors on deoxynivalenol (DON), zearalenone (ZEN) and fumonisin (FUM)-categories are shown; Table S6: Detailed results of FAMD- analysis of the *grain maize hybrid experiment* data set (2011–2013, *n* = 216). Experimental site Cadenazzo (TI) excluded, due to non-congruent hybrids. Association of the different variables on deoxynivalenol, zearalenone and fumonisin-categories are shown; Table S7: General information on the 5 *grain maize hybrid experimental* sites in Switzerland (2011–2013); Table S8: General additional information on the grain maize hybrids used in the *grain maize hybrid experiments*, 2011–2013. SC = single crossed hybrid, TC = Threeway crossed hybrid; Table S9: Categorical and quantitative variables of the grain maize hybrid experiments’ dataset for Cadenazzo. The number of samples is in parentheses; Table S10: Morphologically identified *Fusarium* species throughout the *grain maize hybrid experiments* from the experimental site *Cadenazzo (TI)* 2011–2013, listed according to their relative frequency (%) in the infected grain maize kernels. In addition, mean incidence (%) of the four most prevailing *Fusarium* species in the single years is also indicated (*n* = 33); Table S11: Mean deoxynivalenol, zearalenone and fumonisin contents (µg kg^−1^) as well as the percentages of grain maize samples exceeding the corresponding European guidance values. Data from the *grain maize hybrid experimental site Cadenazzo (TI)*, 2011–2013, *n* = 33. File S1: Supplementary_monitoring_08_10. Excel file containing raw data from commercial grain maize monitoring (2008–2010) in Switzerland, used for statistical analysis. File S2: Supplementary_hybridexperiment11_13. Excel file containing raw data from grain maize experiments (2011–2013), used for statistical analysis.
