# Supplementary material for: A Multi-Year Monitoring of Swiss Grain Maize: Which Cropping Factors Influence Fusarium Species Incidence and Associated Mycotoxins?
Source: Toxins (Basel). 2026 Jan 26;18(2):65. doi: 10.3390/toxins18020065 (PMC12944499; doi:10.3390/toxins18020065)
Supplement: Supplementary file 1 [file toxins-18-00065-s001.zip › Supplementary_data_1.pdf]

Supplementary Material

In order to give more detailed information on the *Swiss commercial grain maize monitoring* conducted during the years 2008-2010 and the *grain maize hybrid experiments* (2011-2013), we provide the following supplementary information, including information on the number and proportion of the samples in the defined mycotoxin classes (Table S1), on the distribution of the samples depending morphologically identified *Fusarium* species frequency, mean *Fusarium* incidence based on seed health tests (SHT) as well as on fungal DNA (Table S2, Table S3), weather conditions during both studies (Figure S1, Figure S2, Table S4), and detailed FAMD-analysis results (Table S5, Table S6). In Figure S3, Spearman rank correlation coefficients between the incidences of fungal species (%) based on SHT and mycotoxins (mg kg^{-1}) from *grain maize hybrid experiments* at the four experimental sites Delley, Ellighausen, Goumoëns and Zurich-Reckenholz (2011-2013) are shown. In Figure S4 the mean *Fusarium* incidence and composition pattern in the *grain maize hybrid experiments* from early, mid-early, and mid-late maturing maize hybrids at the four experimental sites is shown (2011-2013) and in Figure S5 the Log2 fold changes in mycotoxin content (DON, ZEN, and FUM) across maize maturity classes mid-early and mid-late relative to the maturity class early.
In addition, general information about experimental sites and maize hybrid characteristics are given in Table S7 and Table S8. In Supplementary Table S9-S11, detailed information on the excluded maize grain hybrid experimental site Cadenazzo (TI) is given as well as the weather conditions (Figure S6). This experimental site was excluded from the statistical analysis, as maize hybrids were not congruent with hybrids at the other experimental sites.

Supplementary Table S1: Mycotoxin categories used for the statistical analysis of both data sets (2008-2010 *c*ommercial grain maize data set (n=271); 2011-2013 grain maize hybrid experiments) based on mycotoxin levels and EU guidance values (= GVA) for mycotoxin contamination in animal feed and the number of samples in a category as well as their proportion (%).

	Defined range of categories (GVA = guidance value, LOD = limit of detection)	Number of samples in a category and its proportion (%)	
		Commercial grain maize data (*n*=271)	Grain maize hybrid experiments (*n*=216)
DON			
no DON	0 to < 0.22 mg kg^{-1}	51 (19%)	62 (29%)
low DON	≥ 0.22 (LOD ELISA) to < 0.9 mg kg^{-1}	74 (27%)	85 (39%)
medium DON	≥ 0.9 (GVA for pigs) to < 2 mg kg^{-1}	62 (23%)	36 (17%)
high DON	≥ 2 (GVA for calves < 4 months, lambs and kids) to < 5 mg kg^{-1}	51 (19%)	32 (15%)
very high DON	≥ 5 mg kg^{-1} (complementary & complete feeding stuff with above-mentioned exceptions)	33 (12%)	1 (0.5%)
ZEN			
no ZEN	0 to < 0.05 mg kg^{-1}	146 (54%)	171 (79%)
low ZEN	≥ 0.05 (LOD ELISA) to < 0.1 mg kg^{-1}	40 (15%)	25 (12%)
medium ZEN	≥ 0.1 (GVA for piglets/gilts) to < 0.25 mg kg^{-1}	33 (12%)	15 (7%)
high ZEN	≥ 0.25 (GVA sows and fattening pigs) to < 0.5 mg kg^{-1}	21 (8%)	5 (2%)
very high ZEN	≥ 0.5 mg kg^{-1} (GVA for calves, dairy cattle, sheep and goats)	31 (11%)	0 (0%)[1]
FUM			
no FUM	0 to < 0.22 mg kg^{-1}	240 (89%)	186 (86%)
low FUM	≥ 0.22 (LOD ELISA) to < 5.0 mg kg^{-1}	25 (9%)	29 (13%)
medium FUM	≥ 5 (pigs, horses, rabbits and pets) to < 20 mg kg^{-1}	6 (2%)	1 (0.5%)
high FUM	≥ 20 mg kg^{-1} (poultry, calves <4 months, lambs and kids) to < 50 mg kg^{-1}	0 (0%)[1]	0 (0%)[1]
very high FUM	≥ 50 mg kg^{-1} (adult ruminants > 4 months and mink)	0 (0%)[1]	0 (0%)[1]

[1] Not included in statistical analysis as no samples were observed in these categories.

Supplementary Table S2: Morphologically identified *Fusarium* species throughout the *commercial maize grain monitoring* in Switzerland 2008-2010, listed according to their relative frequency (%) in the infected grain maize kernels. Mean incidence (%) based on a seed health test and mean DNA (ng g^{-1}) shown for the four most frequent *Fusarium* species (*n*=271).

Fusarium species	**Relative *frequency* (%) of *Fusarium* species**			**Mean *incidence* (%)** SE /(Median)			**Mean *DNA* (ng g^{-1})** SE /(Median)		
	**2008 (n=91)**	**2009 (n=97)**	**2010 (n=83)**	**2008 (n=91)**	**2009 (n=97)**	**2010 (n=83)**	**2008 (n=91)**	**2009 (n=97)**	**2010 (n=83)**
F. graminearum	40.9	20.9	30.5	5.9 ± 0.7 (3.5)	4.6 ± 0.5 (3.5)	9.4 ± 1.1 (5.5)	9.4 ± 3.7 (3.0)	17.0 ± 4.9 (3.0)	84.7 ± 24.2 (17.4)
F. verticillioides	13.4	22.1	26.2	1.9 ± 0.5 (0)	4.9 ± 1.0 (1.0)	8.1 ± 1.5 (2.0)	1.8 ± 1.3 (0.2)	15.3 ± 5.3 (0.2)	263.3 ± 131 (0.6)
F. subglutinans	20.6	22.6	14.1	2.9 ± 0.7 (0.5)	5.0 ± 0.7 (2.5)	4.3 ± 0.6 (2.0)	0.5 ± 0.05 (0.5)	5.5 ± 1.8 (0.5)	31.3 ± 11.6 (0.5)
F. proliferatum	8.1	12.9	11.8	1.2 ± 0.3 (0.3)	2.9 ± 0.6 (1.0)	3.6 ± 0.7 (0.5)	0.8 ± 0.1 (0.2)	9.6 ± 2.9 (1.8)	37.9 ± 9.7 (4.5)
F. cerealis	3.7	2.0	7.2						
F. poae	2.7	5.7	1.4						
F. equiseti	3.4	3.4	1.7						
F. avenaceum	2.2	3.2	2.3						
F. solani	0.0	0.1	0.02						
F. culmorum	1.5	1.6	1.3						
F. oxysporum	1.4	3.4	1.0						
F. tricinctum	0.2	1.0	0.6						
F. sporotrichioides	0.3	0.3	0.7						
F. sambucinum	0.04	0.0	0.0						
F. semitectum	0.04	0.0	0.0						
F. venenatum	1.3	0.5	0.3						
***Fusarium* spp.**	0.3	0.2	1.6						
**Number of isolates**	1315	2148	2555						
**% infected kernels**	14	22	31						
**Total DNA (*ng g^{-1}*)**							2058	7948	39’302

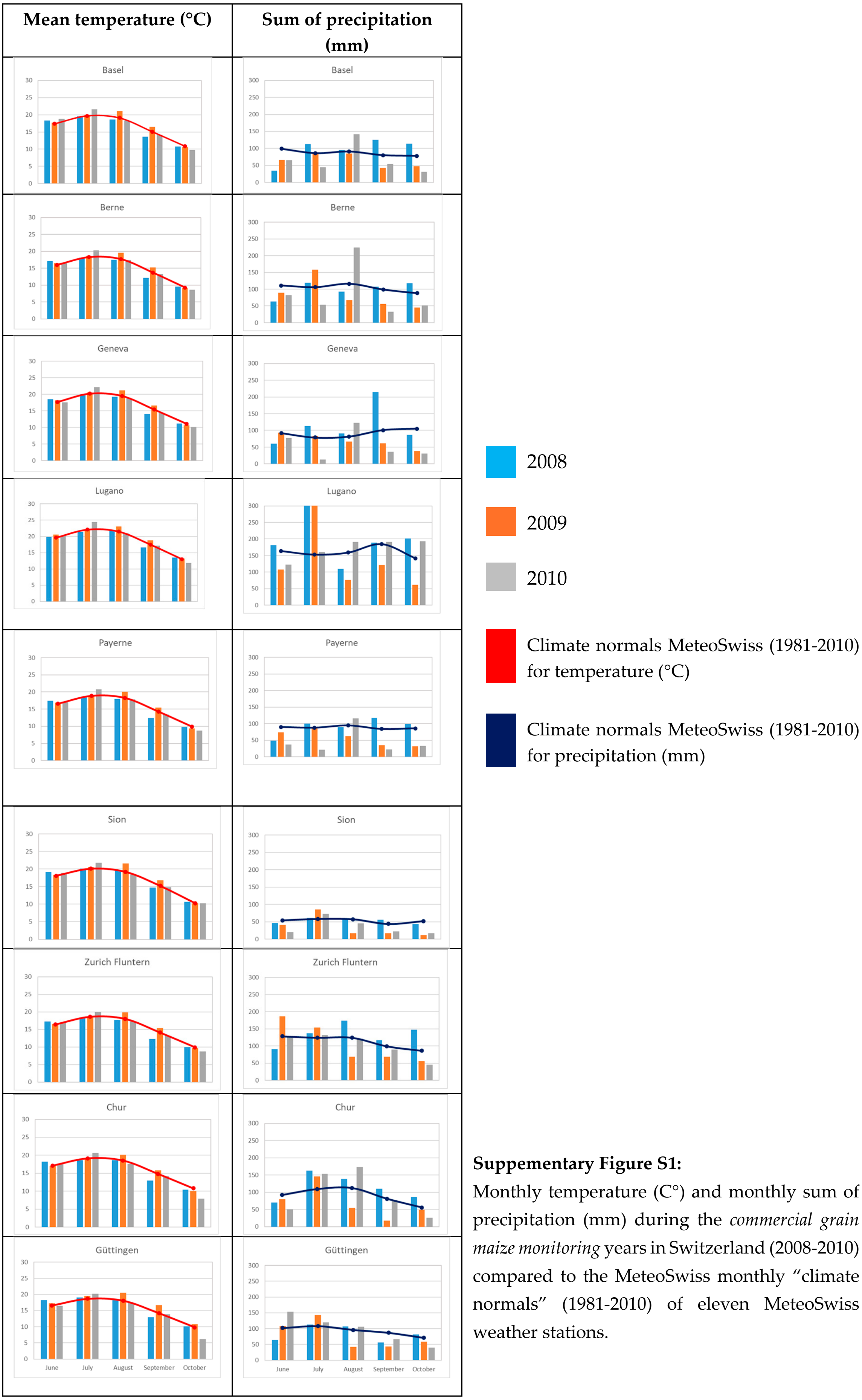

Suppementary Figure S1: Monthly temperature (C°) and monthly sum of precipitation (mm) during the *commercial grain maize monitoring* years in Switzerland (2008-2010) compared to the MeteoSwiss monthly "climate normals" (1981-2010) of eleven MeteoSwiss weather stations.

Supplementary Table S3: Morphologically identified *Fusarium* species throughout the *grain maize hybrid experiments* in Switzerland 2011-2013, listed according to their relative frequency (%) in the infected grain maize kernels. In addition, mean incidence (%) based on a seed health test of the four most prevailing *Fusarium* species in the single years is also indicated (*n*=216). As no overlapping grain maize hybrids were sown at the site Cadenazzo compared with the four sites North of the Alps, this experimental site was excluded from the analysis.

***Fusarium* species**	**Relative frequency (%) of *Fusarium* species**			**Mean *incidence* (%) SE /(Median)**		
	**2011 (n=72)**	**2012 (n=72)**	**2013 (n=72)**	**2011 (n=72)**	**2012 (n=72)**	**2013 (n=72)**
F. graminearum	42.1	73.9	9.4	2.3 ± 0.4 (1.0)	13.1 ± 1.8 (5.0)	2.6 ± 0.4 (1.5)
F. verticillioides	14.5	7.4	62.9	1.3 ± 0.4 (0)	1.3 ± 0.6 (0)	17.6 ± 2.2 (11.5)
F. proliferatum	8.2	3.5	15.1	0.7 ± 0.2 (0)	0.6 ± 0.2 (0)	4.2 ± 0.7 (1.5)
F. subglutinans	15.4	1.5	1.2	1.3 ± 0.3 (0)	0.3 ± 0.1 (0)	0.3 ± 0.1 (0)
F. poae	9.6	4.0	2.8			
F. cerealis	2.2	3.7	3.2			
F. culmorum	2.0	2.0	2.5			
F. avenaceum	1.9	0.0	0.8			
F. equiseti	0.5	0.5	1.2			
F. oxysporum	0.8	0.6	0.6			
F. sporotrichioides	1.1	0.1	0.1			
F. solani	0.6	0.1	0.3			
F. venenatum	0.1	0.1	0.2			
F. tricinctum	0.0	0.1	0.0			
F. semitectum	0.0	0.0	0.1			
***Fusarium* spp.**	0.1	2.7	0.0			
% infected kernels	8.7	17.7	28.0			
Number of isolates	1246	2546	2013			

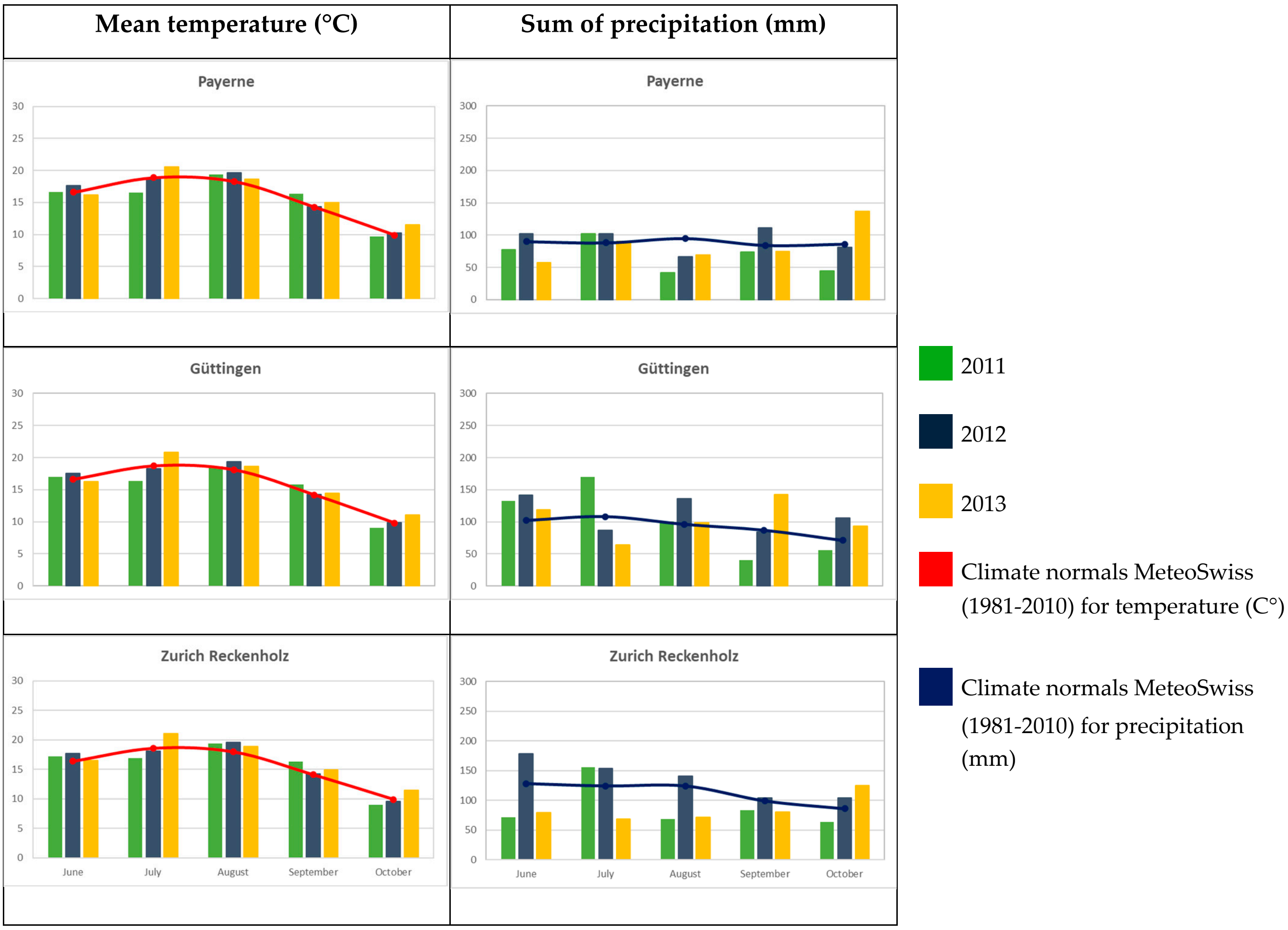

Supplementary Figure S2:
Monthly average temperature (°C) and monthly sum of precipitation (mm) during the *grain maize hybrid experiments* (2011-2013) at the four experimental sites Delley (FR), Ellighausen (TG), Goumoëns (VD), and Zurich-Reckenholz (ZH) compared to the MeteoSwiss monthly "climate normals" (1981–2010) from representative weather stations. Payerne represents experimental sites Delley and Goumoëns, Güttingen represents experimental site Ellighausen and Zurich-Reckenholz represents experimental site Zurich-Reckenholz.

Supplementary Table S4: Difference in the sum of precipitation (mm) in the years 2011 and 2013 compared with 2012 at the *grain maize hybrid experimental sites* Delley, Ellighausen, Goumoëns and Zurich-Reckenholz. Listed MeteoSwiss weather stations are representative for the experimental sites.

experimental site	MeteoSwiss weather station	Sum of precipitation (mm) June-October	Differences (mm) compared with 2012	Climate normals MeteoSwiss (1981-2010)
**Zurich-Reckenholz**	Zurich-Reckenholz			561 mm
2011		401.4	-280.4	
2012		681.8		
2013		425.7	-256.1	
**Ellighausen**	Güttingen			464 mm
2011		498.6	-55.9	
2012		554.5		
2013		516.5	-38	
**Delley & Goumoëns**	Payerne			443 mm
2011		339.9	-123	
2012		462.9		
2013		427.8	-35.1	

Supplementary Table S5: Detailed results of FAMD- analysis of *commercial grain maize monitoring* data set, 2008-2010, (*n*=271). Association of the different factors on deoxynivalenol (DON), zearalenone (ZEN) and fumonisin (FUM)-categories are shown.

DON

categorical variables which are associated with the resulting clusters.

categorical variables (chi-square test)	p-value	df
year	1.046187e-9	8

Mycotoxin category	Cla/Mod	Mod/Cla	Global	p.value	v.test
**"No DON"**					
year=2009	38.144330	72.549020	35.79336	3.379769e-09	5.912001
year=2010	4.819277	7.843137	30.62731	2.627491e-05	-4.203560
**"Low DON"**	no categorical variables associated				
**"Medium DON"**	no categorical variables associated				
**"High DON"**					
year=2009	6.185567	11.76471	35.79336	2.985326e-05	-4.174583
**"Very high DON"**					
year=2009	2.061856	6.060606	35.79336	3.845691e-05	-4.11656

Quantitative variables associated with the formation of the mycotoxin clusters.

Mycotoxin category	v.test	Mean in category	Overall mean	sd in category	Overall sd	p.value
**"No DON"**						
Harvest (Julian day)	-4.666307	286.0784314	293.627306	10.9899118	12.798662	3.066617e-06
ZEN (μg kg^{-1})	-5.358586	3.2759588	4.243728	0.5878869	1.428819	8.387592e-08
qpcr_FG	-5.796238	0.5297538	1.763391	0.8696283	1.683824	6.781909e-09
**"Low DON"**						
ZEN (μg kg^{-1})	-4.850344	3.555575	4.243728	0.9526277	1.428819	1.232475e-06
**"Medium DON"**	no quantitative variables associated					
**"High DON"**						
ZEN (μg kg^{-1})	4.818368	5.113933	4.243728	1.095125	1.428819	1.447369e-06
qpcr_FG	4.152314	2.647145	1.763391	1.483018	1.683824	3.291299e-05
**"Very high DON"**						
ZEN (μg kg^{-1})	9.712286	6.511755	4.243728	1.235185	1.428819	2.672748e-22
qpcr_FG	6.611597	3.582894	1.763391	2.164874	1.683824	3.801953e-11
Harvest (Julian day)	5.885963	305.939394	293.627306	17.646254	12.798662	3.957437e-09
Growing period (days)	3.837791	181.636364	172.708487	18.898161	14.233681	1.241461e-04

ZEN

categorical variables which are associated with the resulting clusters.

categorical variables (chi-square test)	p-value	df
year	3.571918e-07	8

Mycotoxin category	Cla/Mod	Mod/Cla	Global	p.value	v.test
**"No ZEN"**					
year=2009	68.04124	45.20548	35.79336	0.0004782457	3.492654
**"Low ZEN"**	no categorical variables associated				
**"Medium ZEN"**	no categorical variables associated				
**"High ZEN"**	no categorical variables associated				
**"Very high ZEN"**					
year=2010	26.506024	70.967742	30.62731	1.106434e-06	4.871698
year=2009	2.061856	6.451613	35.79336	9.433160e-05	-3.904728

Quantitative variables associated with the formation of the mycotoxin clusters

Mycotoxin category	v.test	Mean in category	Overall mean	sd in category	Overall sd	p.value
**"No ZEN"**						
Sowing (Julian day)	-4.202251	118.472603	120.918819	9.527318	10.337517	2.642743e-05
Harvest (Julian day)	-4.605294	290.308219	293.627306	10.101244	12.798662	4.118831e-06
qpcr_FG	-5.731997	1.219891	1.763391	1.258378	1.683824	9.925482e-09
DON (μg kg^{-1})	-10.007044	6.053888	6.859012	1.095071	1.428763	1.419299e-23
**"Low ZEN"**	no quantitative variables associated					

**“Medium ZEN”**	no quantitative variables associated					
**“High ZEN”**						
DON (µg kg^{-1})[1]	4.035992	8.069858	6.859012	0.8586979	1.428763	5.437218e-05
**“Very high ZEN”**						
qpcr_FG	8.133704	4.082537	1.76339072	2.031197	1.683824	4.163681e-16
DON (µg kg^{-1})	7.922125	8.775671	6.85901163	1.082934	1.428763	2.334848e-15
Harvest (Julian day)	4.949854	304.354839	293.62730627	13.864665	12.798662	7.426924e-07
qpcr_Fvert	4.421510	1.568558	-0.35732539	3.648872	2.572270	9.801355e-06
qpcr_Fsub	4.295051	1.167215	0.00664389	2.395693	1.595734	1.746529e-05
FUM_ppb	4.074340	5.128908	4.29662262	1.781656	1.206347	4.614504e-05
qpcr_Fpro	3.624044	1.577814	0.32649522	2.523431	2.039068	2.900320e-04
Growing period (days)	3.547191	181.258065	172.70848708	16.394271	14.233681	3.893628e-04

FUM

categorical variables which are associated with the resulting clusters.

categorical variables (chi-square test)	p-value	df
Maturity class	0.0001033827	6

**Mycotoxin category**	**Cla/Mod**	**Mod/Cla**	**Global**	**p.value**	**v.test**
**“No FUM”**					
Previous crop = soyabean	20.00000	0.4166667	1.845018	0.0006723650	-3.400607
Maturity class = late	74.24242	20.4166667	24.354244	0.0001160106	-3.854407
**“Low FUM”**	no categorical variables associated				
**“Medium FUM”**					
Maturity class = late	9.090909	100	24.35424	0.0001746169	3.753163
**“High FUM”**	no samples in this category				
**“Very high FUM”**	no samples in this category				

Quantitative variables associated with the formation of the mycotoxin clusters

**Mycotoxin category**	**v.test**	**Mean in category**	**Overall mean**	**sd in category**	**Overall sd**	**p.value**
**“No FUM”**						
qpcr_Fsub	-5.503808	-0.18545159	0.00664389	1.347712	1.595734	3.716741e-08
qpcr_Fvert	-6.440515	-0.71967722	-0.35732539	2.132721	2.572270	1.190685e-10
qpcr_Fpro	-6.835091	0.02165706	0.32649522	1.791070	2.039068	8.195325e-12
**“Low FUM”**						
qpcr_Fpro	4.592613	2.114247	0.3264952	2.144478	2.039068	4.377301e-06
qpcr_Fvert	4.287316	1.747992	-0.3573254	3.432621	2.572270	1.808449e-05
**“Medium FUM”**						
qpcr_Fsub	5.781965	3.738298	0.00664389	1.6352257	1.595734	7.383323e-09
qpcr_Fpro	5.753059	5.071055	0.32649522	0.8776306	2.039068	8.764297e-09
qpcr_Fvert	5.499960	5.364590	-0.35732539	3.3693695	2.572270	3.798772e-08

**"High FUM"**	no samples in this category
**"Very high FUM"**	no samples in this category

Supplementary Table S6: Detailed results of FAMD- analysis of the *grain maize hybrid experiment* data set (2011-2013, n=216). Experimental site Cadenazzo (TI) excluded, due to non-congruent hybrids. Association of the different variables on deoxynivalenol, zearalenone and fumonisin-categories are shown.

DON

categorical variables which are associated with the resulting clusters.

**categorical variables (chi-square test)**	**p-value**	**df**
Previous pre-crop	1.476437e-19	24
Previous crop	6.256211e-09	18
year	6.610926e-08	6
site	4.139624e-07	9

**Mycotoxin category**	**Cla/Mod**	**Mod/Cla**	**Global**	**p.value**	**v.test**
**"No DON"**					
year=2011	45.833333	72.549020	33.33333	1.285999e-06	4.841906
Previous pre-crop = maize	77.777778	7.843137	8.333333	1.295561e-06	4.840434
year=2012	6.944444	9.259259	33.33333	4.592301e-06	-4.582600
**"Low DON"**					
Previous_pre-crop = spring wheat	73.33333	23.65591	13.888889	3.824138e-04	3.551932
Previous pre-crop = pasture/spring wheat	0.00000	0.00000	8.333333	2.221151e-05	-4.241415
**"Medium DON"**	no categorical variables associated				
**"High DON"**					
Previous_pre-crop = pasture/spring wheat	83.33333	45.454545	8.333333	1.369080e-11	6.761142
Previous crop=maize	50.00000	45.454545	13.888889	9.395652e-07	4.903891
year=2012	30.55555	66.666667	33.333333	2.493086e-05	4.215425
site=Reckenholz	33.33333	54.545455	25.000000	8.070346e-05	3.942301
Previous crop= spring wheat	3.030303	6.060606	30.555556	3.482694e-04	-3.576467
site=Delley	0.000000	0.000000	25.000000	2.987675e-05	-4.174404
year=2011	1.388889	3.030303	33.333333	8.833171e-06	-4.443927

Quantitative variables associated with the formation of the mycotoxin clusters

**Mycotoxin category**	**v.test**	**Mean in category**	**Overall mean**	**sd in category**	**Overall sd**	**p.value**
**"No DON"**						
FG	-5.405618	0.08499461	0.1988252	0.07995959	0.1782676	6.458539e-08
ZEN (µg kg^{-1})	-8.221348	0.50878514	2.2854953	1.28340804	1.8294990	2.012289e-16
**"Low DON"**						
FG	-4.049255	0.1422092	0.1988252	0.08197639	0.1782676	5.138093e-05
**"Medium DON"**						
ZEN (µg kg^{-1})	3.844584	3.358119	2.285495	1.202627	1.829499	0.0001207573

**"High DON"**						
FG	8.904839	0.453771	0.1988252	0.2312167	0.1782676	5.346359e-19
ZEN (μg kg^{-1})	5.795954	3.988463	2.2854953	1.0090780	1.8294990	6.793397e-09

```
Link between the cluster variable and the quantitative variables
================================================================
             Eta2      P-value
FG           0.4942262 3.427956e-31
ZEN (μg kg-1) 0.4254659 2.351075e-25
```

ZEN

categorical variables which are associated with the resulting clusters.

categorical variables (chi-square test)	p-value	df

**Mycotoxin category**	**Cla/Mod**	**Mod/Cla**	**Global**	**p.value**	**v.test**
**"No ZEN"**					
year=2011	94.44444	39.76608	33.33333	3.358424e-05	4.147693
**"Low ZEN"**	no categorical variables associated				
**"Medium ZEN"**	no categorical variables associated				
**"High ZEN"**	no categorical variables associated				

Quantitative variables associated with the formation of the mycotoxin clusters

**Mycotoxin category**	**v.test**	**Mean in category**	**Overall mean**	**sd in category**	**Overall sd**	**p.value**
**"No ZEN"**						
DON (μg kg^{-1})	-5.783966	5.921413	6.182124	1.253028	1.288382	7.295966e-09
**"Low ZEN"**	no quantitative variables associated					
**"Medium ZEN"**						
DON (μg kg^{-1})	4.695113	7.692287	6.182124	0.7845634	1.288382	2.664595e-06
**"High ZEN"**						
FUM (μg kg^{-1})	3.573563	5.7874086	3.7413647	2.35229	1.29233921	0.0003521568
Fcul	3.525646	0.1184255	0.0289068	0.12105	0.05731101	0.0004224515

```
Link between the cluster variable and the quantitative variables
================================================================
               Eta2       P-value
DON (μg kg-1) 0.17257676 9.412236e-09
FUM (μg kg-1) 0.08893906 1.873643e-04
```

FUM

categorical variables which are associated with the resulting clusters.

categorical variables (chi-square test)	p-value	df
Previous pre-crop	1.392434e-08	16
year	8.014690e-08	4
Previous crop	1.516051e-07	12

Mycotoxin category	Cla/Mod	Mod/Cla	Global	p.value	v.test
**"No FUM"**					
year = 2011	97.22222	37.634409	33.333333	3.444128e-04	3.579378
Previous pre-crop = potato	44.44444	4.301075	8.333333	1.360941e-05	-4.350070
Previous crop = winter wheat	44.44444	4.301075	8.333333	1.360941e-05	-4.350070
year = 2013	66.66667	25.806452	33.333333	2.116507e-08	-5.602198
**"Low FUM"**					
year = 2013	33.333333	82.758621	33.333333	5.537250e-09	5.830165
Previous pre-crop = potato	55.555556	34.482759	8.333333	9.396318e-06	4.430619
Previous crop = winter wheat	55.555556	34.482759	8.333333	9.396318e-06	4.430619
year = 2011	2.777778	6.896552	33.333333	5.183996e-04	-3.471064
**"Medium FUM"**	no categorical variables associated				
**"High FUM"**	no samples in this category				
**"Very high FUM"**	no samples in this category				

Quantitative variables associated with the formation of the mycotoxin clusters

Mycotoxin category	v.test	Mean in category	Overall mean	sd in category	Overall sd	p.value
**"No FUM"**						
FCe	-3.450069	0.03427346	0.04041821	0.05894773	0.06502669	5.604435e-04
Fpro	-5.318491	0.06078157	0.07755242	0.10276666	0.11512821	1.046311e-07
Fvert	-7.406233	0.10389247	0.15135196	0.18463493	0.23395954	1.299372e-13
**"Low FUM"**						
Fvert	7.134346	0.44041829	0.15135196	0.29022409	0.23395954	9.724839e-13
Fpro	5.101064	0.17925808	0.07755242	0.13358028	0.11512821	3.377494e-07
FCe	3.623415	0.08122311	0.04041821	0.08511404	0.06502669	2.907389e-04
**"Medium FUM"**	no quantitative variables associated					
**"High FUM"**	no samples in this category					
**"Very high FUM"**	no samples in this category					

```
Link between the cluster variable and the quantitative variables
================================================================
            Eta2      P-value
Fvert 0.25710329 1.794232e-14
Fpro  0.13313530 2.464990e-07
Fcul  0.06765221 5.755075e-04
```

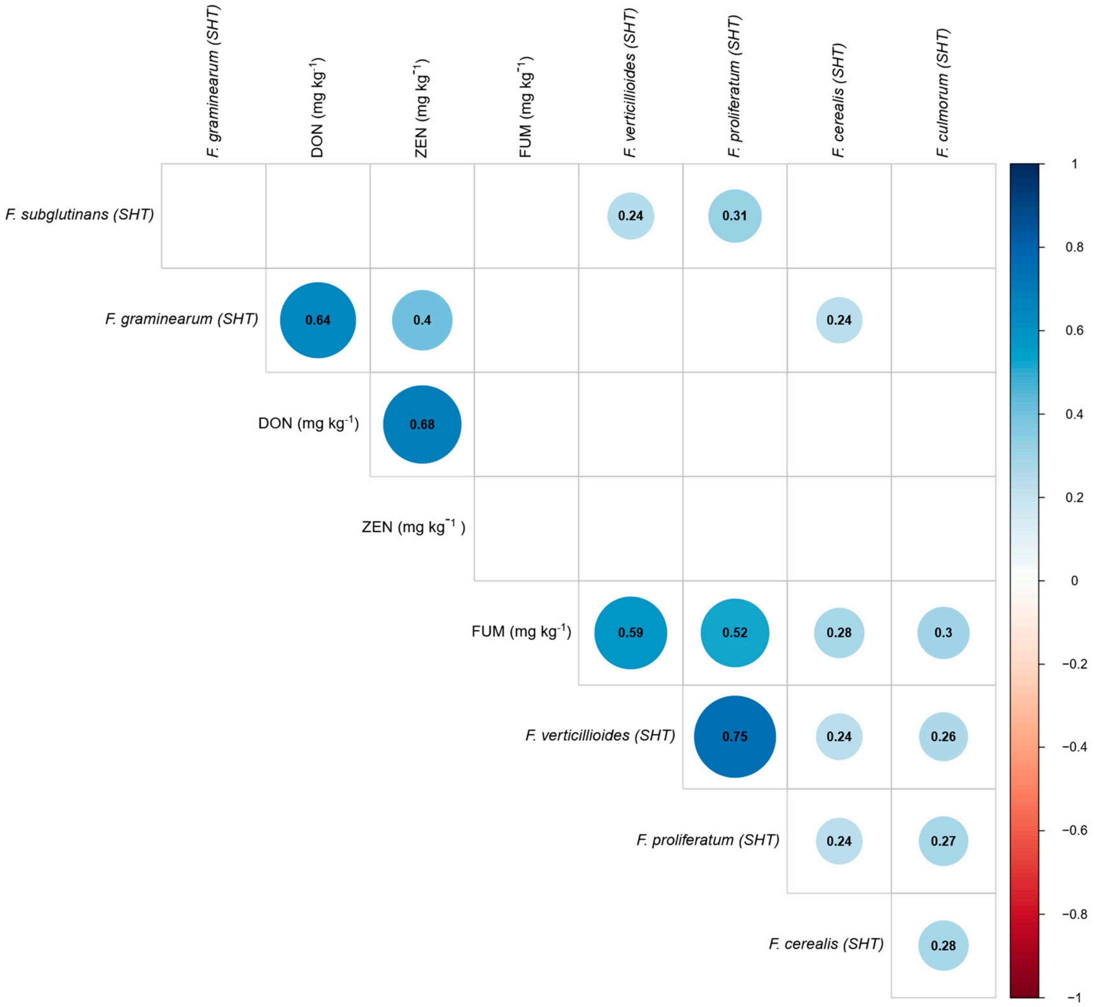

Supplementary Figure S3: Significant Spearman rank correlation coefficients between the incidences of fungal species based on seed health test (SHT) and mycotoxins concentration from *grain maize hybrid experiments* at the four experimental sites Delley, Ellighausen, Goumoëns and Zurich-Reckenholz (2011-2013). The experimental site Cadenazzo was excluded due to non-congruent maize hybrids.

The size and colour of the circle represent the strength of the relationship. The size and colour of the circle represent the strength of the relationship. The axes are arranged based on hierarchical clustering (hclust function in R, version 4.4.3) using the default complete linkage method. DON = deoxynivalenol, ZEN = zearalenone, FUM = fumonisins.

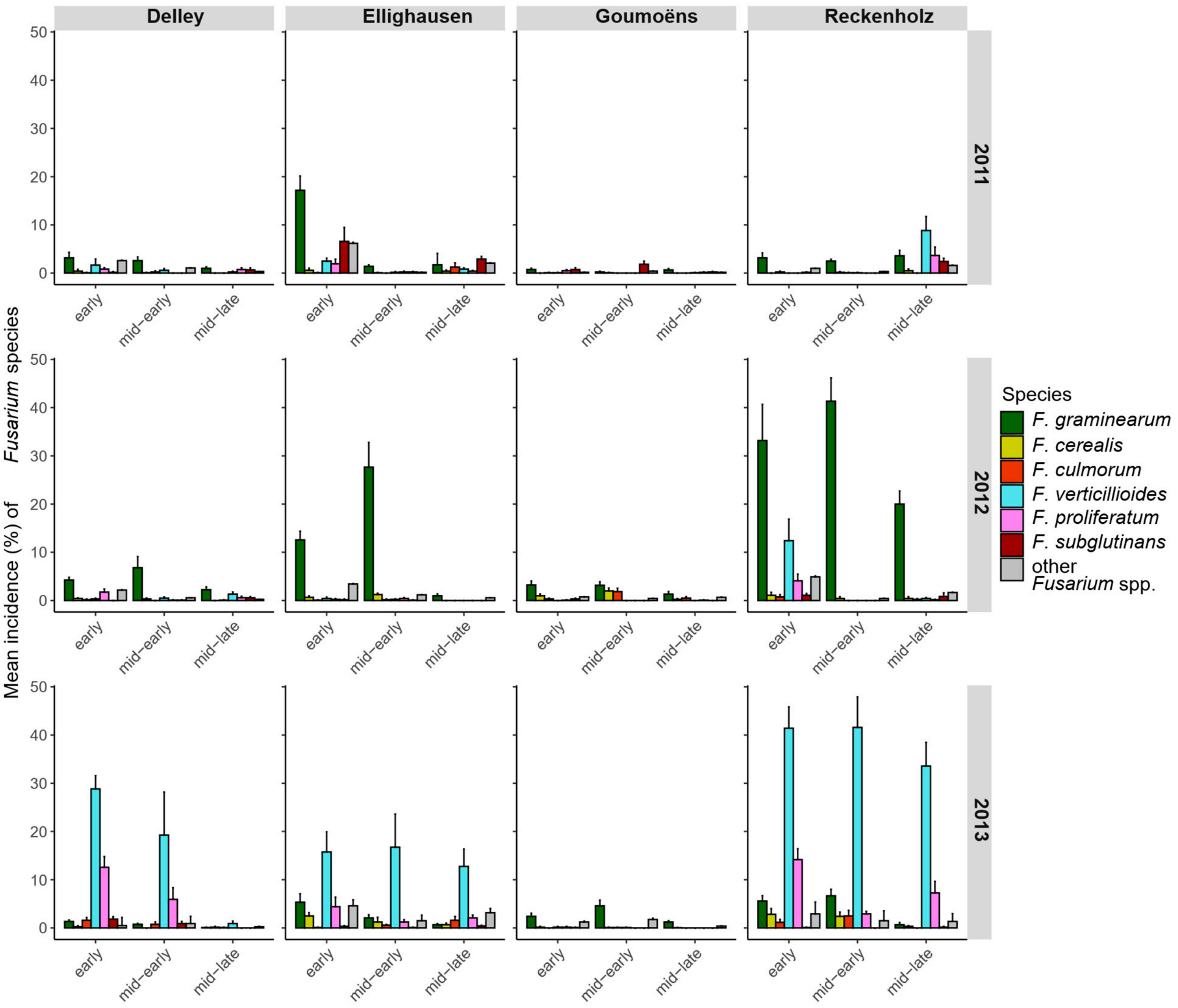

Supplementary Figure S4. Mean *Fusarium* incidence and composition pattern in analysed kernels of the *grain maize hybrid experiments* from early, mid-early, and mid-late maturing maize hybrids throughout three different years (2011, 2012, 2013) at the four experimental sites Delley (FR), Ellighausen (TG), Goumoëns (VD), and Zurich-Reckenholz (ZH), (n = 216). The Cadenazzo (TI) site was excluded as maize hybrids differed from those sown in the other sites.

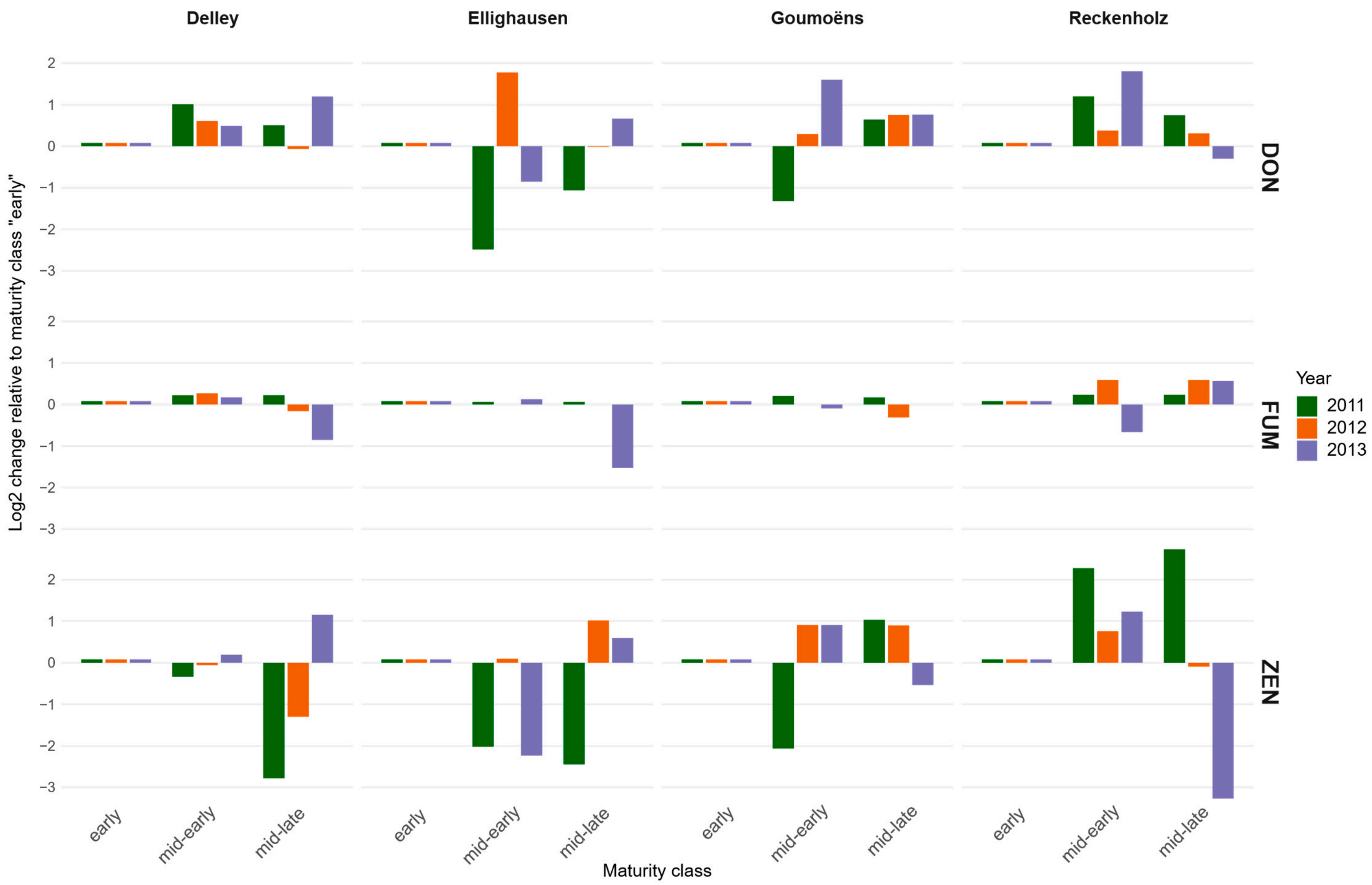

Supplementary Figure S5. Log_2 fold changes in mycotoxin content (DON, FUM, and ZEN) across maize maturity classes mid-early and mid-late relative to the maturity class early (reference, log_2 fold change = 0) at the four experimental sites Delley, Ellighausen, Goumoëns and Reckenholz during 2011-2013. Bars represent mean maturity-driven effects after removal of site and year effects. The absence of significance symbols indicates that no statistically significant differences among maturity classes were detected (Tukey HSD, *P* <

Supplementary Table S7: General information on the 5 *grain maize hybrid experimental* sites in Switzerland (2011-2013).

Experimental site	Height above sea-level (meters above mean sea level)	coordinates	Canton
**Delley**	434	46.9161, 6.9677	Fribourg
**Ellighausen**	517	47.6139, 9.1382	Thurgau
**Goumoëns**	622	46.6500, 6.5987	Waadt
**Zurich-Reckenholz**	440	47.4291, 8.5165	Zurich
**Cadenazzo**	203	46.1604, 8.9310	Ticino

Supplementary Table S8: General additional information on the grain maize hybrids used in the *grain maize hybrid experiments*, 2011-2013. SC= single crossed hybrid, TC= Threeway crossed hybrid.

Delley, Ellighausen, Goumoëns, Zurich-Reckenholz						
**Hybrid**	**Synonym**	**Hybrid type***	**breeder**	**Grain maize**	**Silage maize**	**Registered in ***
Birko	Birko	SC	RAGT	Early		2001
Stuard	KXA 4301	TC	KWS	Early		2006
Laurinio	KXA 8114	TC	KWS	Early		2011
Ricardino	KXA 6123	SC	KWS	Mid-early	Mid-early	2009
NK Cooler	NX 07066	TC	Syngenta	Mid-early		2011
NK Top	NX 04016	SC	Syngenta	Mid-early		2010
DKC3420	EXP 138	SC	DEKALB	Mid-late		2005
Cassilas	KXA 6334	SC	KWS	Mid-late		2010
**Cadenazzo**						
**Hybrid**	**Synonym**	**Hybrid type**	**breeder**	**Grain maize**	**Silage maize**	**Registered in***
Maxxis	RH0130	SC	RAGT	Mid-late		2007
Kassandras	KXA0375	SC	KWS	Mid-late		2013
PR34B39	PR34B39	SC	Pioneer	Early		2010
DKC 5276	DKC 5276	SC	DEKALB	Early		2010
PR38A24	PR38A24	SC	Pioneer	Early		2003

*Hybrids are no longer listed in the hybrid list for Switzerland.

Supplementary information on the excluded *grain maize hybrid experimental* site Cadenazzo (canton Ticino) during 2011-2013

The grain maize hybrid experimental site Cadenazzo (TI) was excluded from the statistical analysis, as the sown grain maize hybrids at this site were not congruent with the grain maize hybrids sown at the other four experimental sites.

Supplementary Table S9: Categorical and quantitative variables of the grain maize hybrid experiments' dataset for Cadenazzo. The number of samples is in parentheses.

**Categorical variables**	
Previous crop	winter wheat (24), pasture (9)
Previous pre-crop	soybean (12), maize (12), pasture (9)
Maturing class	early (15), mid-late (18)
Grain maize hybrid	PR38A24, PR34B39, DKC5276, Maxxis (2011/2012) Kassandras (2013)
Harvest time	Julian day
Site	Cadenazzo (TI)
Harvest year	2011 (12), 2012 (12), 2013 (9)
**Quantitative variables**	
Mycotoxin concentrations	DON mg kg^{-1}, ZEN μg kg^{-1}, FUM mg kg^{-1}
Fusarium species incidence (SHT)[a]	*F. graminearum, F. verticillioides, F. proliferatum, F. subglutinans*

[a]SHT= seed health test

Supplementary Table S10: Morphologically identified *Fusarium* species throughout the *grain maize hybrid experiments* from the experimental site *Cadenazzo (TI)* 2011-2013, listed according to their relative frequency (%) in the infected grain maize kernels. In addition, mean incidence (%) of the four most prevailing *Fusarium* species in the single years is also indicated (*n*=33).

Fusarium species	Relative frequency (%) of *Fusarium* species			Mean *incidence* (%) SE /(Median)		
	**2011 (n=12)**	**2012 (n=12)**	**2013 (n=9)**	**2011 (n=12)**	**2012 (n=12)**	**2013 (n=9)**
F. graminearum	13.7	9.2	15.7	4.3 ± 0.9 (3.5)	4.3 ± 1.2 (2.8)	1.1 ± 0.3 (1.5)
F. verticillioides	13.6	25.3	47.1	4.3 ± 0.8 (4.5)	11.8 ± 1.7 (11.5)	3.2 ± 1.4 (0.5)
F. proliferatum	38.5	51.1	23.1	12.2 ± 2.7 (10.0)	23.8 ± 4.0 (20.8)	1.6 ± 0.7 (0.5)
F. subglutinans	30.6	8.2	3.3	9.7 ± 2.3 (6.3)	3.8 ± 1.1 (3.3)	0.2 ± 0.2 (0.0)
F. poae	0.9	0.7	0.0			
F. cerealis	0.9	0.4	7.4			
F. culmorum	0.0	0.3	0.8			
F. avenaceum	0.1	0.2	0.0			
F. equiseti	0.1	0.3	1.7			
F. oxysporum	0.7	2.8	0.0			
F. sporotrichioides	0.0	0.4	2.5			
F. solani	0.0	0.5	0.0			
F. venenatum	0.0	0.3	0.0			
F. tricinctum	0.0	0.0	0.0			
F. semitectum	0.7	0.0	0.0			
***Fusarium* spp.**	0.8	0.4	0.0			
% infected kernels	31.6	46.6	7.0			
Number of isolates	379.5	559.5	60.5			

Supplementary Table S11: Mean deoxynivalenol, zearalenone and fumonisin contents (µg kg^{-1}) as well as the percentages of grain maize samples exceeding the corresponding European guidance values. Data from the *grain maize hybrid experimental site Cadenazzo (TI)*, 2011–2013, *n* = 33.

	**Deoxynivalenol**		**Zearalenone**		**Fumonisins**	
**Year**	Mean content (mg kg^{-1}) ± 95% confidence Interval (median)	% Samples above guidance value[1]	Mean content (µg kg^{-1}) ±95% confidence Interval (median)	% Samples above guidance value[2]	Mean content (mg kg^{-1}) ±95% confidence Interval (median)	% Samples above guidance value[3]
**2011 (n=12)**	0.28 ± 0.07 (0.22)	8.3	4.6 ± 3.0 (0.9)	0	2.82 ± 0.85 (1.53)	16
**2012 (n=12)**	1.12 ± 0.29 (0.71)	41.7	175.1 ± 92.5 (40.9)	17	11.6 ± 3.44 (5.85)	58
**2013 (n=9)**	1.03 ± 0.19 (0.78)	44.4	0.15 ± 0.06 (0.08)	0	2.38 ± 0.49 (2.34)	0

[1] European guidance value of 0.9 mg kg−1 for DON for complementary and complete feeding stuff for pigs [16]. [2] European guidance value of 0.25 mg kg−1 for ZEN for complementary and complete feeding stuff for sows and fattening pigs [16]. [3] European guidance value of 5 mg kg^{-1} for FUM for complementary and complete feeding stuff for pigs, horses rabbits, and pet animals [16].

At the grain maize hybrid experimental site Cadenazzo (Ticino), *F. verticillioides* and *F. proliferatum* incidence (%) as well as FUM contamination were higher than at the other four experimental sites in all three years (2011–2013). High DON and FUM contamination were observed, even though all fields were ploughed. The year 2012 was the year with the highest DON, ZEN and FUM contamination (Table S10).

**Mean temperature (C°)**	**Sum of precipitation (mm)**

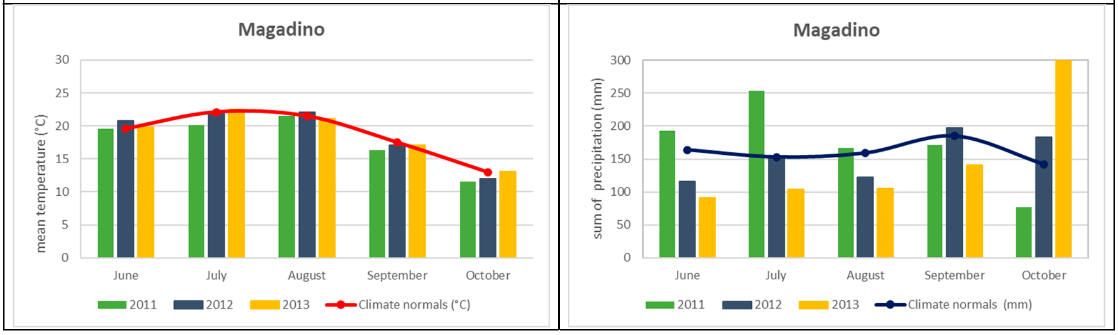

Suppementary Figure S6: Monthly mean temperature (°C) and monthly sum of precipitation (mm) at the MeteoSwiss weather station Magadino during 2011-2013 compared with MeteoSwiss "climate normals" ((1981-2010); red: temperature (°C), blue: precipitation (mm)). The weather station Magadino is representative for the grain maize hybrid experimental site Cadenazzo (Ticino).
